# Substrate Binding Relieves Autoinhibition of the Mammalian GCN2 Kinase

**DOI:** 10.64898/2026.09.09.750423

**Published:** 2026-09-13

**Authors:** Marco A. Catipovic, Rachel Green

**Affiliations:** 1 Department of Molecular Biology and Genetics, Johns Hopkins University School of Medicine; Baltimore, MD, 21205, United States of America; 2 Howard Hughes Medical Institute; Chevy Chase, MD, 20815, United States of America

## Abstract

Cells respond to amino acid starvation and translational stress through the integrated stress response (ISR) kinase GCN2. Current models maintain that GCN2 senses these stresses either by binding deacylated tRNAs that accumulate during amino acid starvation or by recognizing ribosome collisions that arise from perturbation of translation elongation. As tRNA charging is inherently coupled to translation elongation, assessing the relative contributions of these two pathways has proven difficult. To fully separate the roles of these potential agonists, we reconstituted the regulation of GCN2 *in vitro* using recombinant proteins. GCN2 has low basal activity, which is stimulated by the addition of ribosomes or the ribosomal P-stalk. By contrast, deacylated tRNAs fail to activate GCN2 both *in vitro* and *in vivo* and instead counteract activation by the ribosome and inhibit GCN2 auto- and substrate phosphorylation. This inhibition of autophosphorylation requires the presence of GCN2’s substrate, eIF2α, suggesting that the substrate plays a role in kinase regulation. Indeed, eIF2α binding to a newly defined autoinhibitory loop in the GCN2 kinase domain relieves its repression and stimulates autophosphorylation of the kinase’s activation loop. Together, these data redefine the role of tRNAs in the regulation of GCN2 and reveal a mechanism novel to kinases in general by which substrate binding regulates activation loop phosphorylation.

## Introduction

In metazoans, the four ISR kinases, HRI, PKR, PERK, and GCN2, each respond to distinct cellular stressors by phosphorylating the alpha subunit of translation factor eIF2 (eIF2α), leading to inhibition of translation initiation and induction of the transcriptional integrated stress response (ISR)(1–3). GCN2 is activated by amino acid starvation and a variety of ribotoxic stresses that impede translation (4–8). Because of the pleiotropic effects of the ISR, GCN2 dysregulation is associated with a variety of cancers and age-related diseases (9, 10); thus, the mechanism of its activation is of considerable interest.

GCN2 is a large dimeric kinase that harbors an N-terminal RWD domain (11, 12) followed by a degenerate pseudokinase domain (PsK) (13), an active kinase domain (KD) (14, 15), a tRNA synthetase-like domain (HisRS) (16–18), and a ribosome-binding C-terminal domain (CTD) (19). Early studies of GCN2 regulation in yeast identified the HisRS domain as necessary for activation and proposed that deacylated tRNAs that accumulated during amino acid starvation could bind this domain to stimulate the kinase (20–23). However, more recent studies have demonstrated a tRNA-independent mechanism of GCN2 activation by ribotoxic stresses that stall a fraction of ribosomes leading to collisions between stalled and translating ribosomes on the same mRNA (6–8, 24–26). These collisions recruit GCN2 to the ribosome through its obligate cofactor GCN1 (26, 27), and subsequent interactions with the ribosomal P-stalk proteins activate the kinase (28–30).

These models are not mutually exclusive, and separating the contributions of tRNA and ribosomes to this process in cells has proven difficult. Deacylated tRNAs accumulate when cells are starved for amino acids, but the resulting paucity of charged tRNAs also stalls ribosomes and causes collisions (8). Thus, it remains unclear which of these two potential agonists is responsible for kinase activity, or whether there is a cooperative role for coincidental tRNA deacylation and ribosome collisions (24, 26, 31). *In vitro* reconstitution permits independent evaluation of these agonists, but these assays have been fraught. Recombinant yeast GCN2 is constitutively active *in vitro*, preventing assessment of external agonists (23, 32, 33). Mammalian GCN2 is more amenable to reconstitution, and previous studies have shown tRNA to be a relatively weak agonist compared to ribosomes (29, 30).

However, these reconstitutions monitored eIF2α phosphorylation alone and failed to detect the initial GCN2 autophosphorylations that license kinase activity (29, 30). Activation of GCN2, like most kinases, requires phosphorylation of conserved residues in the kinase domain activation loop, T899 and T904 in human GCN2 (34), which induces conformational changes in the kinase domain to make the active site accessible to ATP and eIF2α binding and competent for catalysis (35, 36). While a hallmark of activated GCN2 *in vivo*, previous studies have been unable to detect activation loop phosphorylation of full-length GCN2 *in vitro* and have thus been blind to the regulation of these critical initial events.

Here, using *in vitro* reconstitution with purified components and complementary *in vivo* experiments, we define the contributions of ribosomes and tRNA to the initial activation of GCN2 and subsequent phosphorylation of eIF2α. Surprisingly, we find a crucial role for the substrate itself in the process and thereby describe a novel mechanism of kinase regulation wherein binding of eIF2α to a regulatory loop of GCN2 relieves autoinhibition of the kinase domain.

## Results

### GCN2 Activation by the Ribosomal P-stalk

Previous reconstitutions showed 80S ribosomes to be sufficient agonists of GCN2 *in vitro* (29, 30). Indeed, incubation of recombinant wild-type GCN2 (Fig S1A), but not the catalytically dead D848N mutant, with purified ribosomes leads to autophosphorylation of GCN2 as well as phosphorylation of an established model substrate, the N-terminal domain of eIF2α (Fig S1B, C, D) (37, 38). In fact, the entirety of the ribosome is dispensable; recombinant P-stalk heteropentamer consisting of uL10, P1, and P2 yields similar activation (Fig S1B; Fig 1A, B) (29). These activities can be resolved by western blotting with phospho-specific antibodies, autoradiography to detect ^*32*^P transferred from γ-phosphate-labeled ATP, or by the retarded migration of the phosphorylated eIF2α in an SDS-PAGE gel (Fig 1B; Fig S1D).

Kinetic analyses of these reactions show both auto- and eIF2α phosphorylation by GCN2 to depend on P-stalk concentration (Fig 1C, D, E, F). GCN2 phosphorylation accumulates immediately upon the addition of ATP, whereas substrate phosphorylation exhibits a lag phase characteristic of kinases reliant on autophosphorylation for their activation (Fig S1E) (39). As previously shown (34), and consistent with this dependency, Alanine substitution of either conserved activation loop Threonine abolishes kinase activity (Fig S1F). Analysis of the rate of both auto- and substrate phosphorylation yields the P-stalk concentration dependence of each phosphorylation event (Fig 1G, H). V_0_ of autophosphorylation can be determined from the slope at the initial timepoints, but due to its initial delay phase, the kinetics of substrate phosphorylation are best fit with two-step hypo-exponential functions, reflective of the sequential nature of the process. The maximum of the derivative of these fits serves as a proxy for V_0_. Both the rates of autophosphorylation and substrate phosphorylation share similar dependencies on P-stalk concentration (Fig 1G, H).

### GCN2 Inhibition by tRNA

Deacylated tRNAs, in contrast, do not activate GCN2 *in vitro*. GCN2 has minimal auto- and substrate phosphorylation activity in the absence of P-stalk or ribosomes, visible only by long exposures of autoradiographs (Fig 2A, B). Incubation of the kinase with total purified tRNAs deacylated by alkaline hydrolysis fails to produce increased signal for phospho-eIF2α or phosphor-GCN2 (Fig 2A, B). In fact, tRNAs slightly inhibit the low basal activity of the kinase (Fig 2B). This inhibition by tRNA is even more pronounced in the presence of an agonist, such as the P-stalk. Time resolved kinase assays of GCN2 stimulated by multiple P-stalk concentrations in the presence of a tRNA titration show inhibition of both auto- and substrate phosphorylation with increasing concentrations of tRNAs (Fig 2C, D, E, F, G, H; Fig S2A, B). This inhibition is not attributable to a contaminant in the tRNA preparation, as *in vitro* transcribed tRNAs also inhibit GCN2 (Fig S2C). Nor is it an artifact of kinase assays using only the recombinant N-terminal domain of eIF2α, as phosphorylation of purified endogenous eIF2 trimer is also repressed by tRNA (Fig S2D). This regulation is likely specific to GCN2 amongst the ISR kinases since auto- and substrate phosphorylation by the constitutively active fusion of the PERK kinase domain with GST (40) is not inhibited by tRNA (Fig S2E). Together, these *in vitro* experiments challenge the longstanding notion that deacylated tRNAs activate GCN2.

The dependence of translation elongation on charged tRNA levels in cells complicates testing the direct activation or inactivation of GCN2 by deacylated tRNA *in vivo*. While cellular treatments such as addition of translation elongation inhibitors or depletion of release factors likely stall ribosomes without perturbing tRNA charging, deacylating tRNAs without affecting translation is more challenging. Amino acid starvation or tRNA synthetase inhibitors lead to deacylation of specific tRNAs, but they also stall ribosomes at codons decoded by the missing amino-acyl tRNAs (8, 41, 42). Bacterial amber suppression tRNAs engineered to be bio-orthogonal to mammalian tRNA synthetases provide a mechanism to isolate these processes. These tRNAs are only charged by cognate bacterial synthetases in the presence of specific, frequently non-canonical, amino acid analogs (Fig 3A) (43–45). As GCN2 shows little specificity in its tRNA binding (23), introduction of these tRNAs without their cognate synthetase or amino acid should activate the kinase if deacylated tRNAs alone are sufficient agonists.

However, *in vitro*, these tRNAs inhibit GCN2 activity as well as endogenous tRNAs (Fig S3A). And, while a *Bacillus stearothermophilus* tRNA_Tyr_ (BstY) accumulates in cells when expressed along with its cognate synthetase (RS) in the presence or absence of its bespoke amino acid analog Azido-phenylalanine (AzF) (Fig S3B), it fails to activate GCN2 *in vivo*. GCN2 phosphorylation is not enhanced by transfection of plasmids encoding BstY (Fig 3B), while a canonical GCN2 trigger, starvation for Histidine (His), readily induces GCN2 autophosphorylation (Fig 3B). Furthermore, while activation of the ISR by His starvation inhibits translation initiation leading to accumulation of monosomal ribosomes visible by polysome profiling, expression of BstY does not perturb global translation (Fig 3C).

Several studies have proposed that tRNAs provide additional stimulation to GCN2 activated primarily by interactions with the ribosome, rather than serve as independent agonists (26, 46). However, starvation of cells expressing BstY, in the presence or absence of AzF, produces similar levels of phospho-GCN2 and translation inhibition as starvation of control transfected cells (Fig 3B, C), suggesting that BstY does not synergize with His starvation to induce more potent GCN2 activation.

Most existing models, though, stipulate that the anticodon sequence of the stimulatory deacylated tRNAs must be cognate to the mRNA codon in the ribosomal A site (26, 46). Histidine starvation stalls ribosomes at CAC and CAU codons, clearly not cognate matches for the amber suppressor tRNAs’ CUA anticodon. A better experiment, then, would test the synergistic activation of GCN2 by these tRNAs and ribosomes stalled at UAG amber stop codons (Fig 3D). The small molecule SRI-41315 serves as a molecular glue that triggers the degradation of eRF1, thus trapping ribosomes at stop codons (47, 48). The resulting stalled ribosomes collisions induce GCN2 phosphorylation within 4 hours of drug treatment (Fig S3C, D), but transfection of deacylated amber suppressor tRNAs does not modulate this response (Fig 3E). Treatment of cells expressing BstY or an alternative amber suppressor tRNA derived from an *Escherichia coli* tRNA_Leu_ (EcL) with low or high dosages of SRI-41315 produces similar levels of phosphorylated GCN2 as observed in un- or GFP transfected control cells (Fig 3E). In fact, long exposure of the western blots from control treated cells shows that these exogenously expressed tRNAs inhibit the low basal activity of GCN2, consistent with *in vitro* results (Fig 3E, Fig 2B).

### Mechanism of GCN2 Inhibition by tRNA

Multiple studies have reported that tRNAs bind GCN2 at the tRNA synthetase-homologous HisRS domain, coordinated by the motif 2 (m2) residues conserved from class II tRNA synthetases (18, 20, 21, 23, 29). Surprisingly, the m2 motif is also required for ribosome binding and activation by the ribosomal P-stalk (Fig S4A) (29). Thus, a straightforward hypothesis for GCN2 activation by P-stalk and inhibition by tRNAs is that the two factors compete for this binding site. If tRNA binding is inert and the P-stalk stimulatory, then displacement of bound P-stalk by tRNA would manifest as kinase inhibition. However, several lines of evidence contradict this model. Firstly, while Alphafold3 predicts the binding of the acidic tails of P-stalk proteins to the GCN2 m2 motif and successfully places a tRNA_His_ in the proper position on Histidine tRNA synthetase, it struggles to canonically orient a tRNA_His_ bound to the GCN2 HisRS domain (Fig S4B). Secondly, if direct competition between these factors were regulating the kinase, then the IC_50_ of tRNA would vary with the P-stalk concentration. However, kinase assays in the presence of a five-fold range of P-stalk concentrations show little difference in the inhibitory concentration of tRNA (Fig 2G, H). Lastly, if tRNAs and the P-stalk compete for m2 motif binding, then challenging GCN2-bound P-stalk with tRNAs should elute the P-stalk from GCN2, but a titration of tRNA fails to displace P-stalk from Strep-tagged GCN2 immobilized on beads (Fig S4C).

Rather, the inhibition of basal kinase activity suggests that tRNAs are inhibitory independent of the P-stalk. This intrinsic inhibition is more evident with GCN2 mutants that are active even in the absence of ribosomes or the ribosomal P-stalk. Genetic suppressor screens in yeast for GCN2 mutants that maintained activity in an m2 mutant background identified two mutations in the kinase domain, R794G and F842L, that rendered the kinase constitutively active (49). The corresponding variants of the human protein, S808G and F855L, are active *in vitro* in the absence of P-stalk or ribosomes (Fig S4D). However, both mutants are inhibited by tRNA with a concentration dependence comparable to that of the wild-type kinase (Fig S4E, 2H). Consistent with its initial identification as an m2 suppressor mutant, the S808G variant functions *in vitro* even when combined with m2 mutations in the HisRS domain, though its activity is dampened compared to the variant with S808G alone (Fig S4F). Importantly, despite disruption of the presumed tRNA binding site, this double mutant is still inhibited by tRNA (Fig S4G). Overall, GCN2 inhibition by tRNA appears agnostic to interactions involving its m2 motif, which has long been considered a crucial determinant of tRNA binding. This raises the possibility that GCN2 may be inhibited by a broader range of polyanions beyond deacylated tRNA alone. Indeed, GCN2 can be inhibited *in vitro* with predominantly acylated tRNAs (Fig S4H), RNAs transcribed *in vitro* from scrambled tDNA sequences (Fig S4I), and even the ss- and dsRNA analogs polyA and polyIC (Fig S4J).

### Activation of GCN2 by eIF2α

A general model of kinase function dictates that activation of the kinase domain precedes substrate binding (50). As such, tRNAs’ inhibition of substrate phosphorylation could simply be a manifestation of their inhibition of autophosphorylation during the initial steps of kinase activation. To address this possibility, we simplified our system and focused on the autophosphorylation of GCN2 and its inhibition by tRNA, leaving out the substrate which seemed unlikely to contribute to these early steps. Strikingly, though, tRNAs added to GCN2 and P-stalk in the absence of eIF2α fail to inhibit the autophosphorylation of GCN2 (Fig 4A, B, C, D; Fig S5A), despite being a potent repressor of this step when the substrate is present (Fig 4E, 2H). These observations raise the unprecedented possibility that GCN2 activation requires interactions with eIf2α.

If GCN2 is indeed activated by its substrate, then its autophosphorylation should be sensitive to the concentration of eIF2α. Time resolved kinase assays with a titration of eIF2α directly test this proposition (Fig 4F, G; Fig S5B). As anticipated, the efficiency of substrate phosphorylation depends on the concentration of substrate, and accordingly, these experiments provide an estimate of GCN2’s K_m_ for eIF2α-NTD (Fig S5C, D). However, the rate of GCN2 autophosphorylation also scales significantly with the concentration of eIF2α-NTD or purified endogenous eIF2 trimer (Fig 4H; S5B, E), with an EC_50_ for eIF2α-NTD near 2 μM (Fig 4I). These data are consistent with the substrate potentiating the kinase’s activation.

Given this role for eIF2α in activating GCN2, it is possible that the observed inhibition by RNA could result from RNA displacing bound substrate from the kinase. GCN2 and eIF2α co-immunoprecipitation experiments test this hypothesis. Indeed, eIF2α binds GCN2 *in vitro*, even in the absence of P-stalk or ATP (Fig S5F), and competition with a titration of tRNA partially displaces the bound eIF2α-NTD (Fig 4J). Together, these data suggest that eIF2α is an activator of GCN2 and that RNAs inhibit GCN2 activation by disrupting a regulatory interaction necessary for autophosphorylation of the kinase.

### GCN2 Determinants of Activation by Substrate

Based on its homology to other ISR kinases, GCN2 likely binds eIF2α through the C-terminal lobe of its kinase domain to catalyze its phosphorylation. Crystal structures of the related kinase PKR bound to eIF2α-NTD, as well as Alphafold3 predictions of the GCN2 KD-substrate complex, both show the S1 domain of eIF2α cradling the αG helix of the kinase domains (Fig S6A) (38). Intriguingly, in some crystal structures of the substrate-free GCN2 kinase domain, the dephosphorylated activation loop (yellow) lies over the αG helix in a position that would clash with substrate binding (Fig S6A) (15). These interactions between its activation loop and αG helix could maintain GCN2 in an inhibited conformation. In this model, kinase activation would be facilitated by substrate binding which displaces this loop, freeing it for autophosphorylation, and activating the kinase.

The activation loop of GCN2 is significantly longer than those of the other ISR kinases, especially PKR (Fig S6B), which renders it uniquely able to engage the αG helix. If this interaction is indeed repressive, chimeric GCN2 constructs with the endogenous activation loop substituted with those of HRI, PKR, or PERK should be primed for autophosphorylation independent of substrate binding and thus should see no stimulation in the presence of eIF2α. However, while these constructs have overall reduced activity relative to wild-type GCN2, autophosphorylation of each of these chimeras is still accelerated by eIF2α (Fig S6C), suggesting that the interaction between the activation loop and the αG helix is not responsible for substrate-mediated stimulation of GCN2 autophosphorylation.

Furthermore, a previously described mutation of Thr924 within the αG helix of yeast GCN2 impairs substrate phosphorylation without perturbing autophosphorylation (51). Mutation of the corresponding Thr943 in human GCN2 to Ala or Asp also disrupts substrate phosphorylation, but the autophosphorylation of these variants is only slightly reduced and still depends on the substrate (Fig S6D). As autophosphorylation of kinase mutants with an αG helix incapable of coordinating substrate phosphorylation are still stimulated by the substrate, the αG helix likely does not play a significant role in the activation of GCN2 by eIF2α.

While mutation of Thr943 abrogates eIF2α phosphorylation, it does not impair substrate binding to GCN2 (Fig S6E), raising the possibility of a second eIF2α binding site within GCN2. Each of the four ISR kinases harbors a loop insertion between the β4 and β5 strands in the N-terminal lobe of the kinase domain (Fig S7A). These loops are predicted to be largely unstructured and show little homology to one another (Fig S7A, B). The loop in PERK is rich in S/T residues (Fig S7B, C) and when phosphorylated, these residues provide an additional interface for eIF2α binding separate from the canonical αG helix (40). This interaction is proposed to improve the efficiency of catalysis by locally concentrating the substrate (40).

The human GCN2 kinase domain insertion loop has a lower density of S/T residues but is modestly enriched in D/E residues (Fig S7B). These negative charges could recruit eIF2α analogously to the phosphorylated Ser/Thr residues of PERK. If substrate recruitment to this loop stimulates GCN2 activity, autophosphorylation of a variant lacking the loop should be refractory to eIF2α stimulation. Indeed, autophosphorylation of a GCN2 variant lacking a 125 amino acid stretch containing much of the loop (positions 663–788; ΔKDloop) is not stimulated by eIF2α and exhibits reduced inhibition by tRNA (Fig 5A; Fig S8A, B), consistent with the insertion loop mediating substrate activation of GCN2. Strikingly, this deletion renders the kinase constitutively active, exhibiting both auto- and substrate phosphorylation even in the absence of P-stalk or ribosome agonists (Fig 5A).

This *in vitro* constitutive activity of the KDloop deletion is also observed *in vivo*. Endogenous GCN2 is autophosphorylated at Thr899 upon starvation for amino acids such as Histidine (Fig 3B, 5B). Wild-type GCN2 expressed from a transfected plasmid in a GCN2 KO HEK293T cell line at near endogenous levels also undergoes autophosphorylation upon His starvation, but a transfected kinase-dead D848N variant does not, and the constitutive S808G variant is phosphorylated at Thr899 even in complete media (Fig 5B). Remarkably, the ΔKDloop variant also displays activation loop phosphorylation in both starved and unstressed cells (Fig 5B), consistent with its high basal activity *in vitro*. Notably, both constitutively active variants are expressed at lower levels than the wild-type or kinase-dead proteins (Fig 5B); their basal activity likely induces the integrated stress response which represses their own translation.

Together, these data suggest the GCN2 insertion loop is an auto-repressive element whose effects are relieved by substrate binding. Two motifs within the loop stand out as potential regulatory elements. The first is a highly conserved segment including a β-strand predicted by AlphaFold to complement β4 and extend the 5-stranded β-sheet of the kinase domain N-terminal lobe (positions 695–723; Fig 5C, D). The second motif constitutes an acidic patch of consecutive Asp and Glu residues (position 732–740), which is present in all *Chordata* species and comprises the largest negatively charged patch within the loop (Fig 5C, D). The GCN2 variant lacking the first motif (Δ695–723) has high constitutive activity and its autophosphorylation is less responsive to both eIF2α and tRNA (Fig 5E, F; Fig S8C), suggesting that the conserved β-strand is repressive. In contrast, deletion of the acidic patch (Δ732–740A) renders the kinase less active, implying it is not a negative regulator like the first motif. However, loss of the acidic patch still abrogates both stimulation by the substrate and inhibition of autophosphorylation by tRNA (Fig 5E, F; Fig S8C), consistent with a role for this patch in relieving the repression of the neighboring β-strand. Finally, co-immunoprecipitation experiments demonstrate that the acidic patch is required for high affinity binding of eIF2α to GCN2, whereas the conserved β-strand is dispensable (Fig 5G). Altogether, these mutagenesis experiments establish the kinase domain insertion loop as a regulatory element that binds eIF2α substrate through its acidic patch to alleviate the repression of the adjacent conserved β-strand motif.

### eIF2α Determinants of GCN2 Activation

Kinases are often regulated by feedback mechanisms whereby phosphorylation of a substrate potentiates the activity of the kinase (52). This feedback accelerates the kinases’ response to triggering agonists. The activation of GCN2 by eIF2α could be an example of a direct feedback mechanism to increase potency of the GCN2 response to low levels of ribosome collisions. In this model, a small population of active GCN2 would phosphorylate eIF2α. Phospho-eIF2α could then activate further GCN2 proteins, rapidly amplifying the initial agonism by the ribosome.

GCN2 phosphorylates eIF2α on S52 within an exposed loop spanning positions 47–66 of the S1 domain (Fig 6A, B). Were substrate phosphorylation required to stimulate the kinase’s autophosphorylation, then a phospho-null S52A variant of eIF2α would be ineffective, whereas a phosphomimetic S52D variant would more potently activate GCN2. However, the S52A variant instead stimulates GCN2 autophosphorylation equivalently to the WT protein, whereas the S52D variant does not (Fig 6B, C), suggesting that direct feedback through substrate phosphorylation does not regulate the activation of GCN2. In fact, eIF2α S52D also binds to GCN2 with lower affinity than the WT or S52A variant (Fig 6D), consistent with phosphorylation mediating the substrate’s release from the kinase, not its recruitment.

The residues directly following S52 in the eIF2α phosphorylated loop are highly basic (Fig 6B). As an acidic patch on GCN2 is required for substrate stimulation, and the introduction of a single acidic residue adjacent to this motif (S52D) is sufficient to disrupt substrate binding and stimulation of GCN2, then neutralization of this basic patch might be predicted to have similar effects. Indeed, mutation of eIF2α R53–55 to Ala oblates both GCN2 binding and stimulation of autophosphorylation (Fig 6B, C, D), as does deletion of the entire region from residues 49–59 (Fig 6B, C, D). These data suggest that interactions between the basic stretch surrounding S52 and the acidic patch in the GCN2 insertion loop could be responsible for the stimulation of kinase autophosphorylation by eIF2.

In fact, this region alone is sufficient to stimulate GCN2 autophosphorylation. Previous reports have shown that a peptide consisting of the phosphorylated loop, lacking the rest of the S1 domain, can serve as a substrate *in vitro*, albeit with a much higher K_m_ for GCN2 than eIF2α-NTD or eIF2 trimer (53). Indeed, GCN2 incubated with P-stalk phosphorylates a synthesized peptide consisting of residues 47–66 of eIF2α (Fig 6E; Fig S9A, B, C). This peptide also potentiates the autophosphorylation of GCN2 on its activation loop, albeit with an EC_50_ much higher than for the eIF2α-NTD (Fig 6F, G; Fig S9C, D, E). Like with the full NTD, GCN2 stimulation by the substrate peptide requires basic charges adjacent to S52; both a S52p phosphorylated peptide and a charge neutralized R53–55A variant fail to enhance GCN2 autophosphorylation, whereas the S52A variant is partially activating (Fig S9A, B). Thus, the unphosphorylated S52 loop of eIF2α is both necessary and sufficient to stimulate the autophosphorylation of GCN2.

## Discussion

Altogether, these data suggest a model whereby the kinase insertion loop of GCN2 functions as an auto-repressive element, likely through the binding of its conserved predicted β-strand to the β-sheet in the N-terminal lobe of the kinase domain. eIF2 binding to this loop, potentially templated by charge interactions between positive residues surrounding S52 of eIF2α and a negatively charged stretch within the insertion loop, releases the autoinhibition, licensing GCN2 activation. RNAs disrupt the binding of eIF2α to this acidic motif and prevent GCN2 autophosphorylation. This regulation is orthogonal to activation by the ribosome or ribosomal P-stalk, which is communicated primarily through the HisRS and CTD domains (Fig 7).

The mechanism by which the conserved β-strand within the GCN2 insertion loop represses kinase activity is unclear. Crystallized constructs of the GCN2 kinase domain all lack the flexible insertion loop (14, 15), so its effect on the domain’s conformation is uncertain. A recent study, however, proposed that a GCN2 dimer phosphorylates its activation loop only after parallel back-to-back alignment of the kinase domains mediated in part by a “hydrophobic cradle” formed between the β4s of both N-terminal lobes (54). As this is the same interface that the insertion loop β-strand is predicted to bind, an attractive model would be that the insertion loop prevents kinase domain dimerization, and thereby autophosphorylation, until substrate binding to the loop displaces the β-strand and allows interaction between the N-terminal kinase domain lobes (Fig 7).

Such allosteric regulation of a kinase by its own substrate is rare, but not unprecedented. For instance, Aurora Kinases B and C (AURKB/C) complex with the centromeric protein INCENP, which is itself an Aurora kinase substrate (55). Phosphorylation of the C-terminal “IN box” of INCENP increases the autophosphorylation activity of AURKB/C as well as their activity towards other substrates (56, 57). Likewise, binding of several different G-protein Receptor Kinases (GRKs) to activated GPCRs induces a conformational change to allosterically stabilize the active state of the kinase (58, 59). However, these mechanisms seem distinct from the substrate relief of the auto-repressive β-complementation within GCN2. Instead, the GCN2 β-sheet extension structurally resembles that of the Nuclear Factor κB-Inducing Kinase (NIK), whose N-terminal kinase domain extension contributes two additional β-strands to the kinase domain β-sheet beyond β4(60, 61). However, while the conserved β-strand in GCN2 is inhibitory, the N-terminal extension of NIK is activating, as it packs against the N-terminal kinase lobe and is thought to stabilize its active conformation (60, 61). As such, it seems possible that the mechanism of substrate-mediated activation of GCN2 through relief of its autorepression may be unique to this kinase.

In principle, such substrate-dependent activation could increase the specificity of the kinase for its substrate. A potential GCN2 substrate would have to bind first the insertion loop to activate the kinase and then the active site to be phosphorylated. However, phosphorylation by all four ISR kinases is already considered highly specific for eIF2α, requiring the S1 domain of eIF2α to bind the αG helix of the kinase domain to enforce stringent substrate selection^33,44^. It is unclear why GCN2 would involve an extra layer of regulation, or whether the auto-repression of GCN2 may instead have other roles.

For instance, the GCN2 insertion loop may function as a platform for external regulatory factors to modulate kinase activity. Phosphorylation of the analogous loop in PERK recruits eIF2 to the kinase to increase the local concentration of substrate (40). However, competition between other cellular factors and the substrate for these binding sites can regulate and restrict the kinase (62). For example, the mitochondrial OMM protein ATAD3A binds this loop and represses PERK in the vicinity of mitochondrial membranes (63). This presents a tempting parallel for regulation of GCN2 by RNAs. Though deacylated tRNAs have long been considered agonists of the kinase, they seem largely inert *in vivo* and are repressive *in vitro*. Indeed, if deacylated tRNAs were sufficient to activate GCN2 *in vivo*, they would render the kinase nearly constitutively active as, despite the efficiency of acylation, the enormous abundance of tRNA is predicted to leave around 5 μM tRNA deacylated at any given time, even in cells replete with amino acids (64, 65). Rather, RNAs, including deacylated tRNAs, inhibit GCN2 by blocking eIF2 binding to the GCN2 insertion loop. In cells, this may dampen the basal activity of GCN2, preventing spurious eIF2 phosphorylation.

Endogenously, this repression could be lifted in part by the activities of GCN1, ABCF3 (GCN20 in yeast), or conformational rearrangements of GCN2 induced by binding collided ribosomes. Indeed, the regulation outlined here is only a piece of the emerging picture of GCN2 activation in cells. GCN2 is basally inhibited not only by its own auto-repression and RNAs, but also potentially by translation elongation factors, such as eEF2, which impede its access to the P-stalk, and interactions with free 60S subunits (26, 66). *In vitro*, the absence of these inhibitors permits GCN2 activation independent of GCN1 or ribosome collisions (29). However, in cells these positive regulatory factors are likely required to strip away the layers of inhibition and recruit GCN2 to its ideal agonist, a ribosome with an empty A site and an accessible P-stalk (26, 30, 67).

It is also possible that RNAs exert their inhibitory activity *in vitro* not by binding to GCN2, but by binding to eIF2α. The GTP-bound eIF2 trimer binds tRNA_iMet_ with high affinity and specificity. However, in the absence of GTP and tRNA_iMet_, the complex can bind other RNAs with lower affinity (68), as can the isolated α subunit (69–71). If promiscuous RNA binding to eIF2α displaced it from the GCN2 insertion loop *in vitro*, this would manifest as inhibition of GCN2 by RNA. In cells, the eIF2 trimer exists in both a ternary complex bound with tRNA_iMet_ and GTP, as well as a tRNA-free GDP-bound form. If tRNA-bound eIF2 is inhibitory *in vitro*, this implies that the tRNA_iMet_-bound ternary complex may be an insufficient co-activator of GCN2 *in vivo* and that the GDP-bound eIF2 trimer lacking tRNA would instead be a preferred substrate. As phosphorylated eIF2 inhibits eIF2B, its own GEF required for tRNA binding, this would produce a feedback mechanism whereby continued activity of GCN2 and inactivation of eIF2B builds up an increasingly larger pool of tRNA-free eIF2, which in turn further activates GCN2.

Clearly, the role of the substrate as an active participant in GCN2 activation has been underappreciated. While further studies will establish the ramifications for this regulation *in vivo*, the careful *in vitro* reconstitution here has demonstrated that GCN2 is basally autoinhibited by its kinase domain insertion loop and that allosteric activation by its own substrate relieves this repression to unleash full kinase activity. The kinase insertion loop also offers an alternative pharmacological target to inhibit or agonize the kinase *in vivo* (62). Existing ISR inhibitors tend to suffer from “paradoxical activation” whereby high concentrations of these ATP-competitive drugs activate related kinases (72, 73). Peptides or small molecules that disrupt or reinforce the binding between eIF2 and GCN2 could inactivate or potentiate GCN2, respectively, without perturbing the activity of the other ISR kinases, which appear to lack an auto-repressive insertion loop. Considering the disease implications of ISR dysregulation, such meticulous control of a kinase poised to impact cell fate seems appealing. Its discovery here represents an important step in understanding control of GCN2 and the ISR and introduces a new paradigm for kinase regulation in general.

## Supplementary Material

1

Materials and Methods

Figures S1 to S9

References (*74–81*)

## Figures and Tables

**Figure 1.**
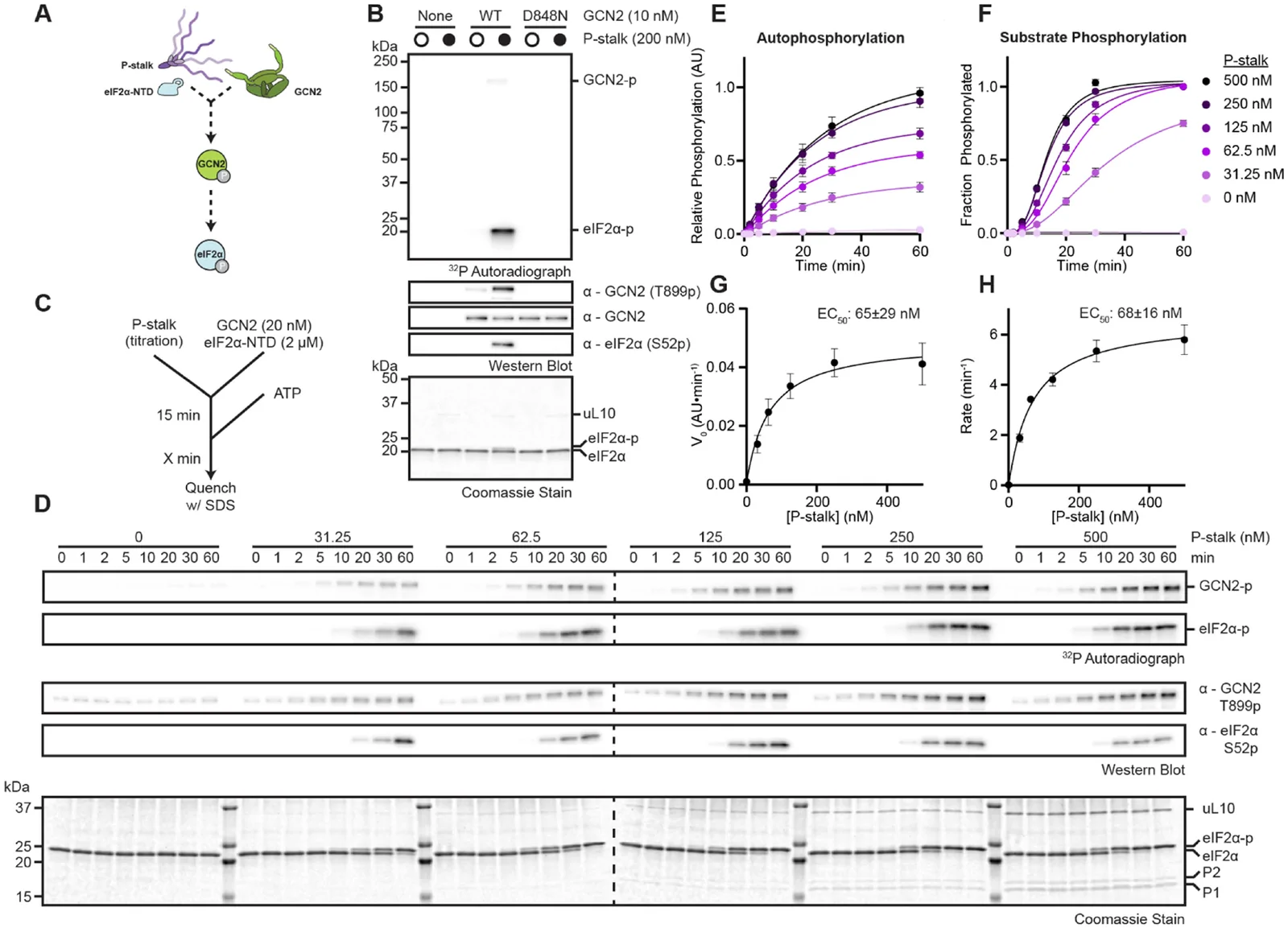
GCN2 Activation by the Ribosomal P-stalk. **(A)** Schematic of a kinase assay using recombinant GCN2 (green), P-stalk (purple), and eIF2α-NTD (light blue). **(B)** Coomassie stain, autoradiograph, and immunoblots with the indicated antibodies of kinase assays as in (A) in the presence or absence of 200 nM P-stalk with 10 nM wild-type (WT) or kinase-dead D848N GCN2 variants and 2 μM eIF2α-NTD. **(C)** Flow chart of kinetic experiment based on kinase assays schematized in (A). **(D)** Coomassie stain, autoradiographs, and immunoblots with the indicated antibodies of kinetic kinase assays with a titration of P-stalk as outlined in (C). **(E)** Intensities of autophosphorylation autoradiographs as in (D) plotted and fit with single exponential functions. Error bars represent S.E.M. of 4 independent replicates. **(F)** As in (E), but with substrate phosphorylation autoradiographs normalized to fraction of phosphorylated eIF2α-NTD visible by Coomassie staining at 60 min. **(G)** V_0_ in (E) as a function of P-stalk concentration fit with a 3-term agonist dose response curve (see Methods). Error bars represent S.E.M. of 4 different replicates. Error on reported EC_50_ is the standard error of the fit. **(H)** As in (G), but with V_Max_ of substrate phosphorylation in (F).

**Figure 2.**
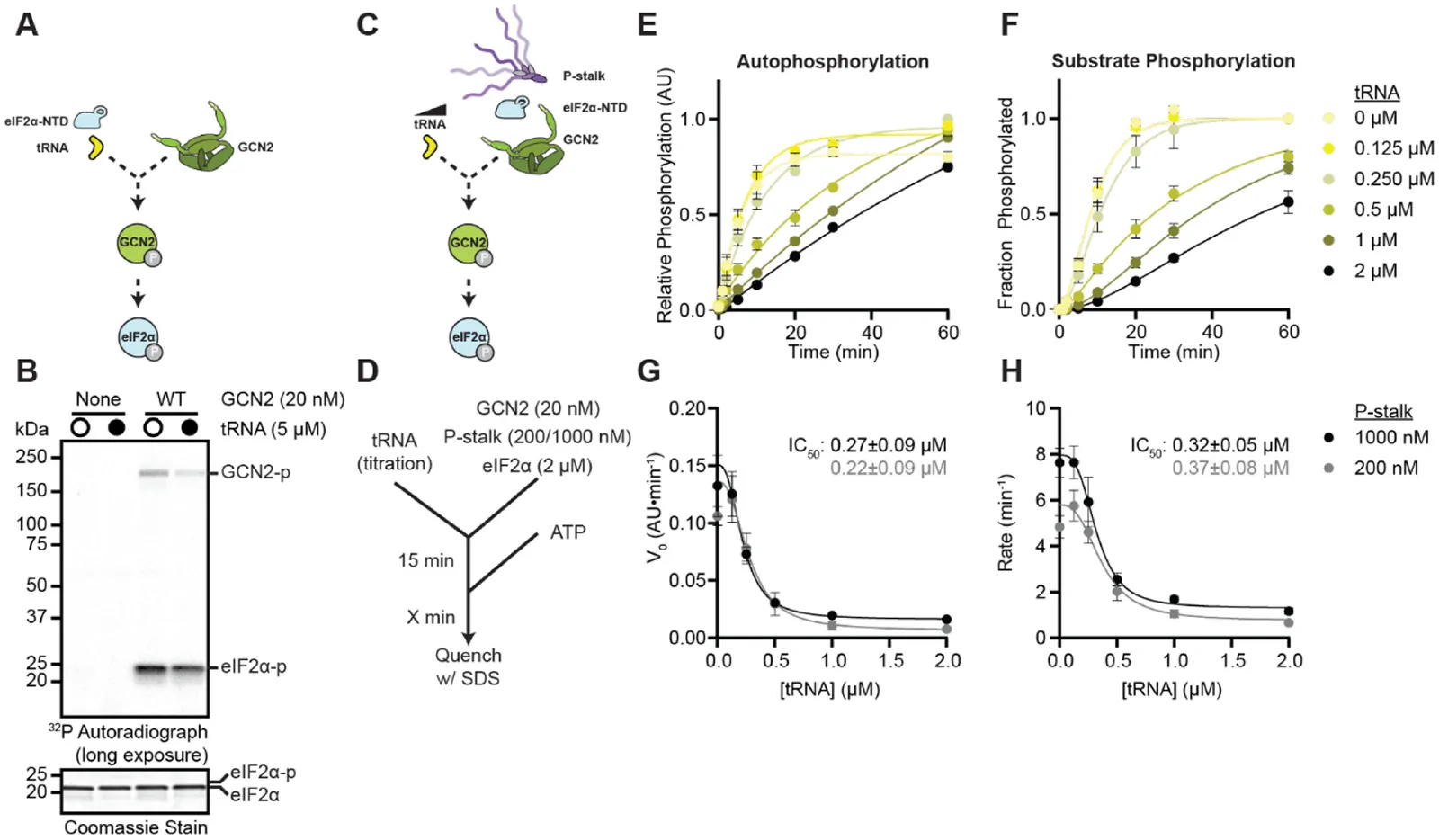
GCN2 Inhibition by tRNA. **(A)** Schematic of a kinase assay using recombinant GCN2 (green), eIF2α-NTD (light blue), and purified deacylated pig liver tRNA (yellow). **(B)** Coomassie stain and autoradiograph (72 h exposure) of kinase assays as in (A) in the presence or absence of tRNA. **(C)** Schematic of a kinase assay as in (A) with the addition of P-stalk (purple). **(D)** Flow chart of kinetic experiment based on kinase assays schematized in (C). **(E)** Intensities of autophosphorylation autoradiographs from experiments as in (D) with 1000 nM P-stalk (Figure S2A) plotted and fit with single exponential functions. Error bars represent S.E.M. of 2 independent replicates. **(F)** As in (E) but with substrate phosphorylation autoradiographs normalized to fraction of phosphorylated eIF2α-NTD visible by Coomassie staining at 60 min. **(G)** V_0_ in (E) as a function of P-stalk concentration fit with a 4-term inhibitor dose response curve (see Methods). Error bars represent S.E.M. of 2 different replicates. Error on reported IC_50_ is the standard error of the fit. **(H)** As in (G), but with V_Max_ of substrate phosphorylation in (F).

**Figure 3.**
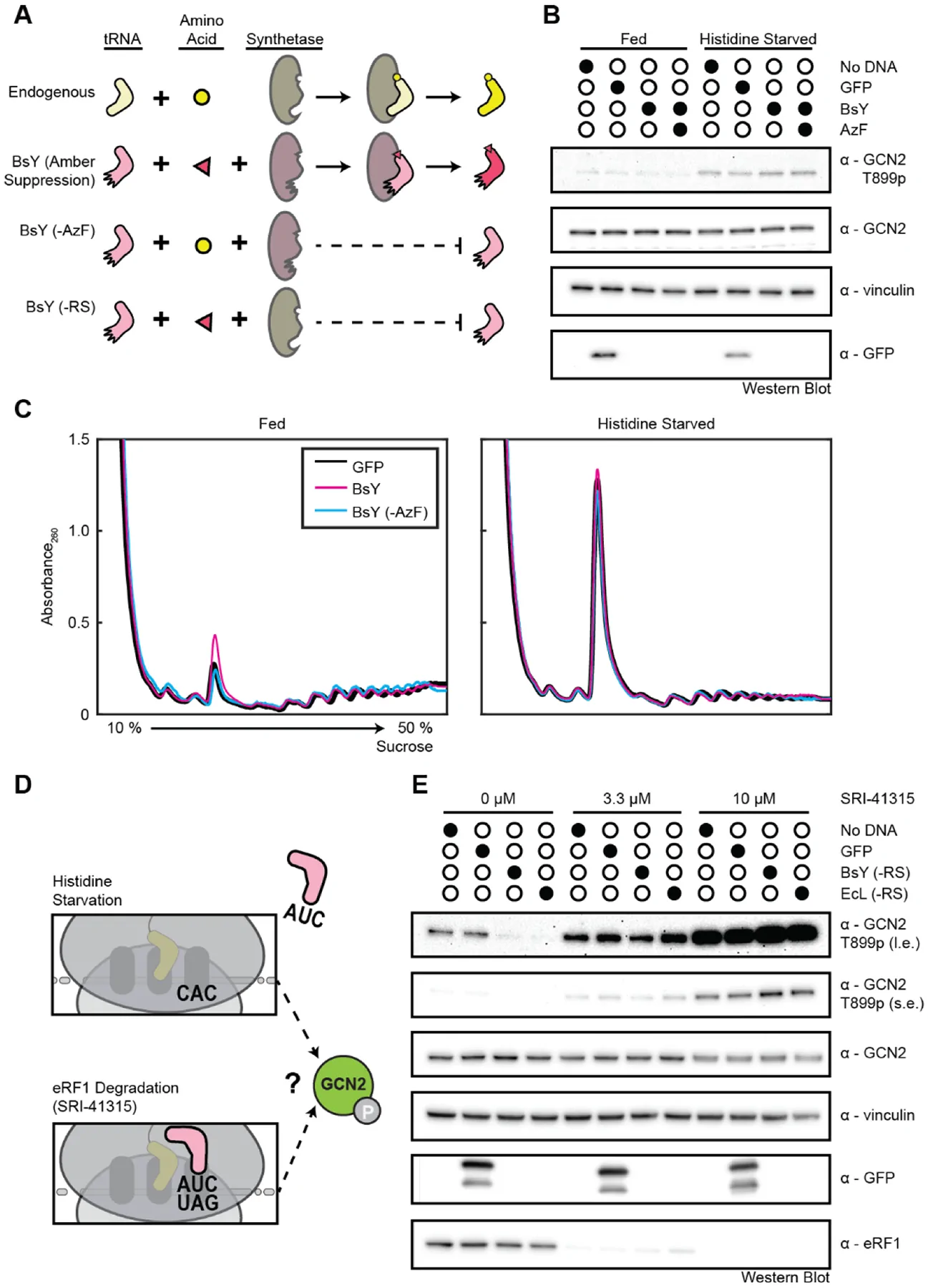
Deacylated tRNAs do not Activate GCN2 *In vivo*. **(A)** Charging of endogenous and amber suppressor tRNAs in cells. Amber suppressor tRNAs (BsY) are only charged in the presence of their cognate synthetase (RS) and their intended amino acid (AzF). **(B)** Immunoblots of GCN2 activity in ΔPKR HEK293T cells transfected with no DNA, GFP (pFD042), or BsY amber suppression plasmids (pIRE4-Azi) in the presence or absence of Azido-Phenylalanine (AzF) grown on complete media or starved of Histidine for 1 h. **(C)** Sucrose gradients from ΔPKR HEK293T transfected as in (B) in the presence or absence of AzF grown on complete media or starved of Histidine for 1 h. **(D)** Histidine starvation stalls ribosomes at codons non-cognate to amber suppressor tRNA anticodons. The eRF1 degrader SRI-41315 stalls a fraction of ribosomes at UAG stop codons cognate to amber suppressor anticodons. **(E)** Immunoblots of GCN2 activity in ΔPKR HEK293T cells transfected with no DNA, GFP (pFD042), BsY amber suppression plasmid lacking the RS (pMC389), or EcL amber suppression plasmid lacking the RS (pMC390) and treated with the indicated dosage of SRI-41315 for 4 h to induce eRF1 degradation.

**Figure 4.**
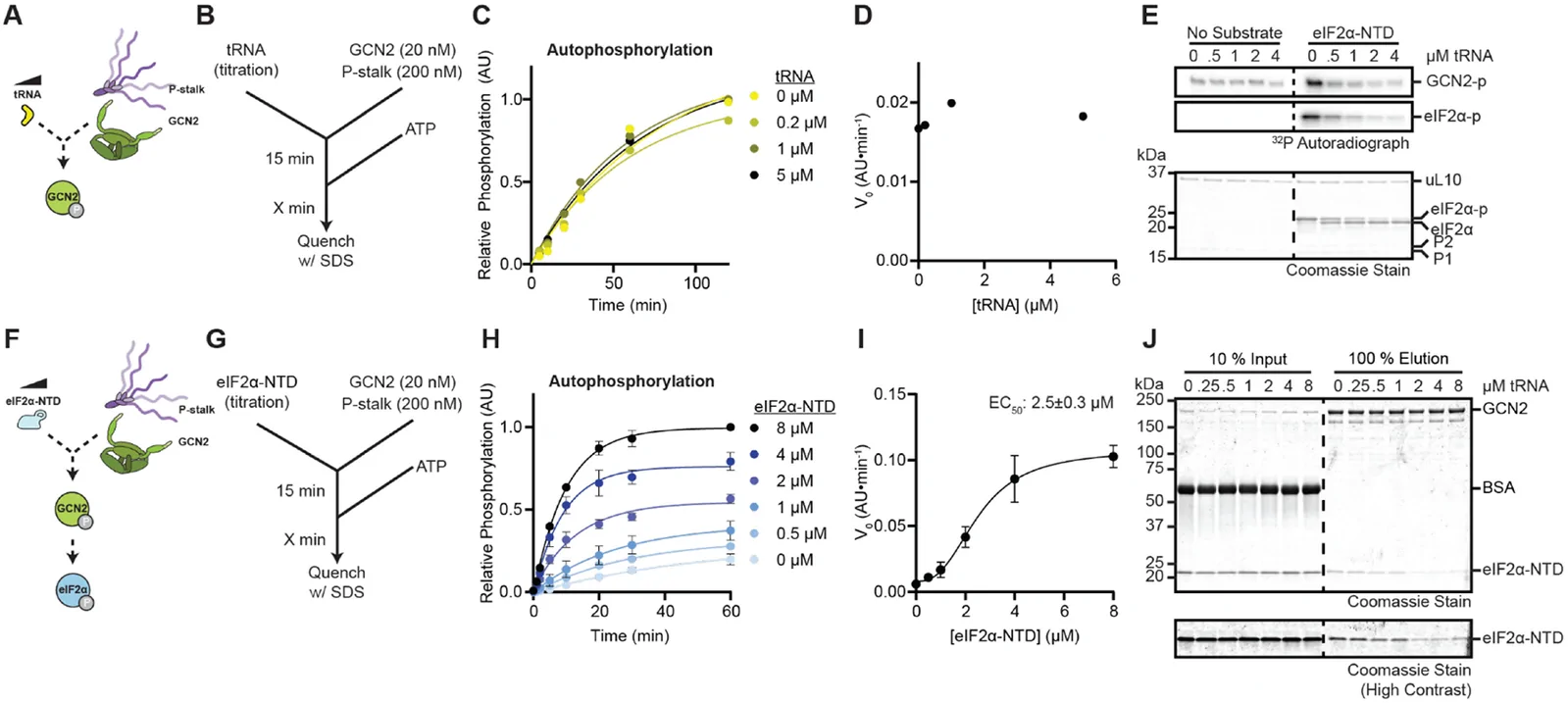
Activation of GCN2 by eIF2α. **(A)** Schematic of kinase assay using recombinant GCN2 (green), P-stalk (purple), and purified deacylated pig liver tRNA (yellow). **(B)** Flow chart of kinetic experiment based on kinase assays schematized in (A). **(C)** Intensities of autophosphorylation autoradiographs from experiments as in (B) with 200 nM P-stalk (Fig S5A) plotted and fit with single exponential functions. **(D)** V_0_ in (C) as a function of tRNA concentration. **(E)** Coomassie stain and autoradiographs from kinase assays as schematized in (A) run for 30 min with 20 nM GCN2 with a titration of tRNA in the presence or absence of 2 μM eIF2α-NTD. **(F)** Schematic of a kinase assay as in (A) but with a titration of eIF2α-NTD (light blue) in place of tRNA. **(G)** Flow chart of kinetic experiment based on kinase assays schematized in (F). **(H)** Intensities of autophosphorylation autoradiographs from experiments as in (G) with 1000 nM P-stalk (Figure S5B) plotted and fit with single exponential functions. Error bars represent S.E.M. of 2 independent replicates. **(I)** V_0_ in (H) as a function of eIF2α-NTD concentration fit with a 4-term agonist dose response curve (see Methods). Error bars represent S.E.M. of 2 different replicates. Error on reported EC_50_ is the standard error of the fit. **(J)** Coomassie stains from co-immunoprecipitation experiments with 200 nM Strep-tagged GCN2 and 2 μM eI2Fα-NTD in the presence of a titration of tRNA. The region including eIF2α-NTD is displayed at two separate contrast levels for clarity.

**Figure 5.**
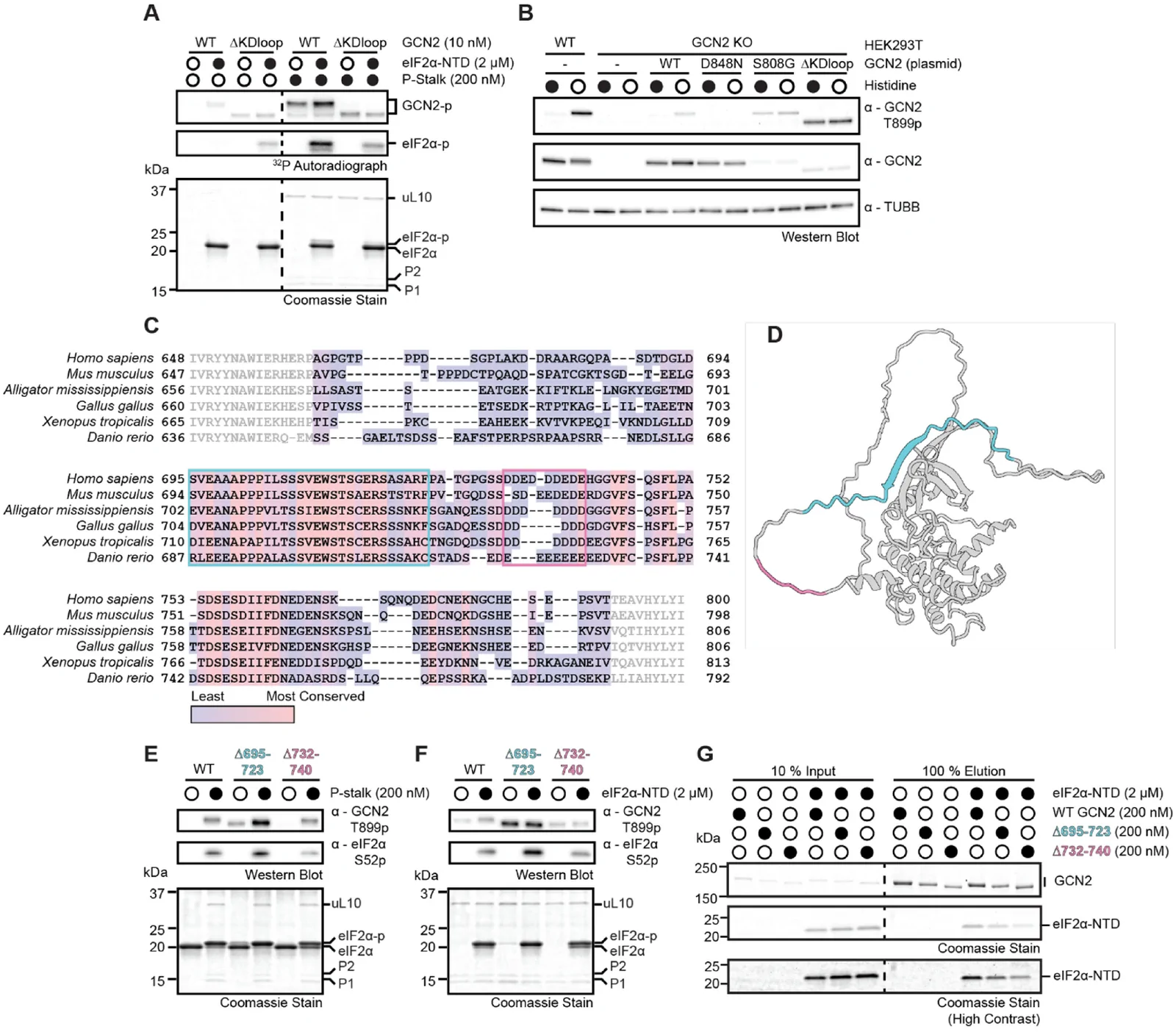
GCN2 Determinants of Activation by Substrate. **(A)** Coomassie stain and autoradiographs of kinase assays with wild-type (WT) and kinase insertion loop deletion (ΔKDloop) GCN2 variants in the presence of 200 nM P-stalk and 2 μM eIF2α-NTD, as indicated. **(B)** Immunoblots of GCN2 activity in wild-type (WT) or ΔGCN2 HEK293T cells transfected with no DNA, wild-type (WT) GCN2 (pMC256), kinase dead GCN2 D848N (pMC574), constitutive active GCN2 S808G (pMC576), or GCN2 ΔKDloop (pMC581) grown on complete media or starved for Histidine for 1 h. **(C)** GCN2 kinase domain insertion loop from species of 6 representative phyla drawn from a Clustal Omega alignment of 818 *Chordata* species. Conservation across all 818 species is indicated in purple to pink color gradient. Insertion loop residues are indicated by darker font. The conserved region containing a predicted β-strand (695–723 in hsGCN2) is boxed in blue. The region containing an acidic patch (732–740) is boxed in pink. **(D)** Structural model of the GCN2 kinase domain (585–1016, *hs*GCN2; Alphafold3). The conserved β-strand and acidic patch are colored as in (C). **(E)** Coomassie stain and immunoblots with the indicated antibodies of kinase assays with 20 nM of the indicated GCN2 variants and 2 μM eIF2α-NTD in the presence or absence of 200 nM P-stalk. **(F)** As in E in the presence or absence of 2 μM eIF2α-NTD. **(G)** Coomassie stains of co-immunoprecipitation experiments with 200 nM of the indicated GCN2 variants in the presence or absence of 2 μM eIF2α-NTD. The region including eIF2α-NTD is displayed at two separate contrast levels for clarity.

**Figure 6.**
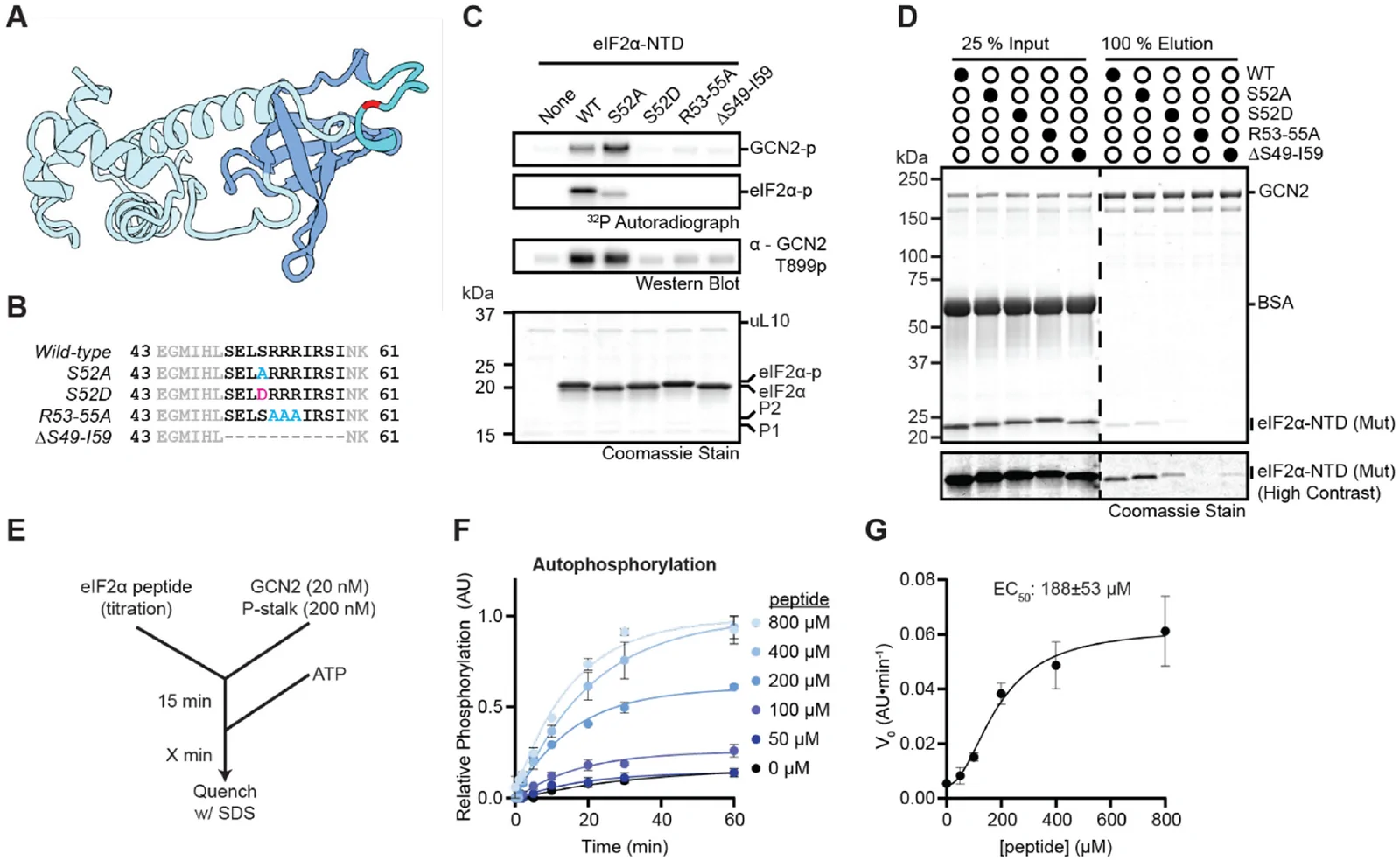
eIF2α Determinants of GCN2 Activation. **(A)** Structural model of the eIF2α-NTD (2–185, *hs*EIF2S1; Alphafold3). The S1 domain is shaded in darker blue, the phosphorylated loop (S49-I59) in cyan, and S52 in red. **(B)** Variants of eIF2α-NTD with Aspartate substitutions colored in magenta and Alanine substitutions colored in cyan. **(C)** Coomassie stain, autoradiographs, and immunoblots with the indicated antibodies of kinase assays with 20 nM GCN2, 200 nM P-stalk, and 4 μM of the indicated eIF2α-NTD variants. **(D)** Coomassie stains of co-immunoprecipitation experiments with 200 nM of GCN2 and 2 μM of the indicated eIF2α-NTD variants. The region including eIFα-NTD is displayed at two separate contrast levels for clarity. **(E)** Flow chart of a kinase experiment as in Fig 4G, but with eIF2α peptide in place of eIF2α-NTD. **(F)** Intensities of autophosphorylation autoradiographs from experiments as in (E) with 200 nM P-stalk (Figure S9C) plotted and fit with single exponential functions. Error bars represent S.E.M. of 2 independent replicates. **(G)** V_0_ in (F) as a function of eIF2α-NTD concentration fit with a 4-term agonist dose response curve (see Methods). Error bars represent S.E.M. of 2 different replicates. Error on reported EC_50_ is the standard error of the fit.

**Figure 7.**
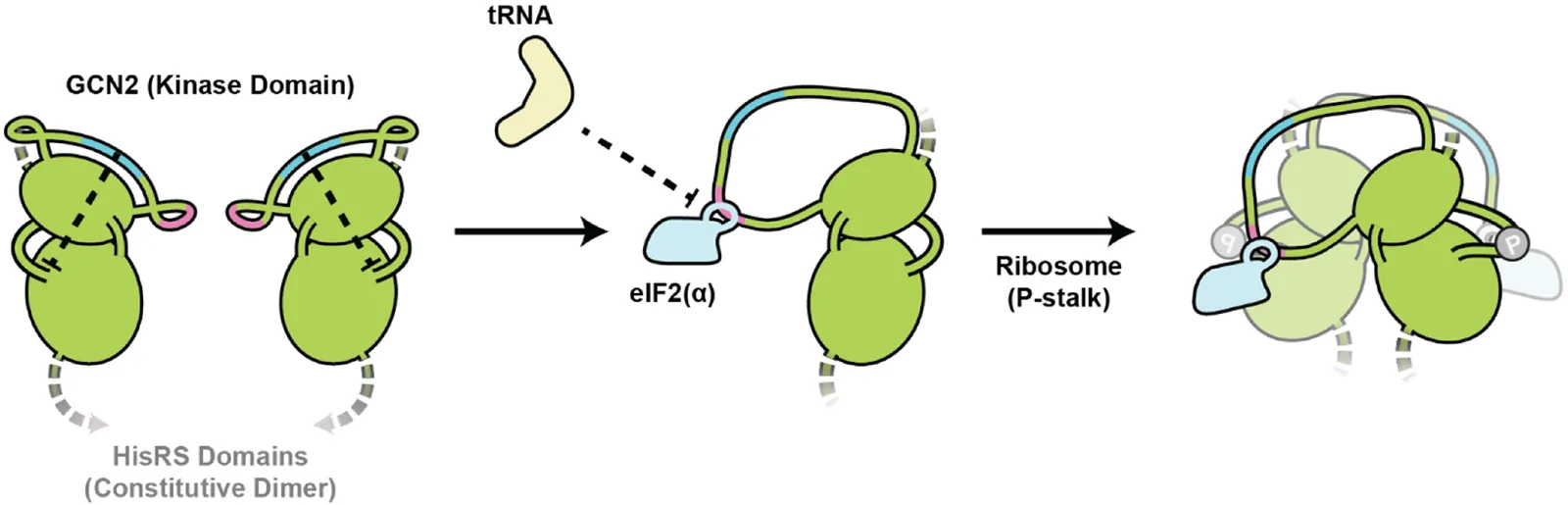
Model of GCN2 Activation by Substrate. GCN2 is constitutively dimerized through interactions in its HisRS and CTD domains. In the absence of substrate and ribosomes, complementation between the conserved β-strand (blue) in the kinase domain insertion loop and β4 in the N-terminal lobe represses GCN2 autophosphorylation. eIF2 binding to the acidic patch displaces the β-strand but is antagonized by tRNA. Once freed from auto-repression, the kinase domains can dimerize in the presence of P-stalk or ribosome to promote autophosphorylation.

## Data Availability

All materials are available upon request from RG.
