# Supplementary material for: Substrate Binding Relieves Autoinhibition of the Mammalian GCN2 Kinase
Source: bioRxiv. 2026 Sep 13:2026.09.09.750423. Preprint. [Version 1] doi: 10.64898/2026.09.09.750423 (PMC13622680; doi:10.64898/2026.09.09.750423)

bioRxiv preprint doi: https://doi.org/10.64898/2026.09.09.750423; this version posted September 13, 2026. The copyright holder for this preprint (which was not certified by peer review) is the author/funder, who has granted bioRxiv a license to display the preprint in perpetuity. It is made available under a CC-BY 4.0 International license.

Supplementary Materials:

Materials and Methods:

DNA Constructs

Overexpression Plasmids for Protein Purification

Plasmid	Description	Source
pCDNA3.1	Mammalian CMV Expression Vector	Thermofisher, V79020
pMC256	pcDNA3.1 backbone. StrepII tag - 3C Site - hsGCN2	This Study
pMC426	pMC256 w/ S808G	This Study
pMC429	pMC256 w/ F1143L, R1144L (m2)	This Study
pMC431	pMC256 w/ D848N	This Study
pMC432	pMC256 w/ T899A, T904A	This Study
pMC435	pMC256 w/ F855L	This Study
pMC449	pMC256 w/ S808G, F1143L, R1144L	This Study
pMC519	pMC256 w/ Δ663-788 (ΔKDloop)	This Study
pMC535	pMC256 w/ HRI Tloop	This Study
pMC536	pMC256 w/ PKR Tloop	This Study
pMC537	pMC256 w/ PERK Tloop	This Study
pMC538	pMC256 w/ T943A	This Study
pMC540	pMC256 w/ T943D	This Study
pMC555	pMC256 w/ T899A	This Study
pMC556	pMC256 w/ T904A	This Study
pMC572	pMC256 w/ Δ695-723	This Study
pMC573	pMC256 w/ Δ732-740	This Study
pAI173	pMultiBac backbone. FL P-stalk. GST-TEV-uL10/P1/P2	Roger Wiliams Lab (*29*)
p9Bc	pET C-terminal TEV His6 cloning vector	Addgene, 48285
pMC257	p9Bc backbone. eIF2αΔCTD - His6. ΔT185 - D315. Solubility optimized (*74*)	This Study
pMC530	pMC257 w/ S52D	This Study
pMC532	pMC257 w/ S52A	This Study
pMC547	pMC257 w/ R53A, R54A, R55A	This Study
pMC570	pMC257 w/ ΔS49-I59	This Study
pGTf2	pACYC backbone. GroES, GroEL, Trigger Factor	Takara, 3340

Transfection Plasmids for In vivo Experiments

bioRxiv preprint doi: https://doi.org/10.64898/2026.09.09.750423; this version posted September 13, 2026. The copyright holder for this preprint (which was not certified by peer review) is the author/funder, who has granted bioRxiv a license to display the preprint in perpetuity. It is made available under a CC-BY 4.0 International license.

Plasmid	Description	Source
pIRE4-Azi	Synthetase tRNA pair for Azido-phenylalanine (AzF) incorporation at amber codons in mammals	Addgene, 105829
pANAP	Synthetase tRNA pair for Anap incorporation at amber codons in mammals,	Addgene, 48696
pMC389	pIRE4-Azi w/ Synthetase ORF deleted	This Study
pMC390	pANAP w/ Synthetase ORF deleted	This Study
pFN24K	pFN24K Mammalian strongly compromised CMV Expression Vector	Promega, G3780
pMC505	pFN24K backbone. 3xFlag tag - hsGCN2	This Study
pMC574	pMC505 w/ D848N	This Study
pMC576	pMC505 w/ S808G	This Study
pMC581	pMC505 w/ Δ663-788	This Study
pFD042	pFC15K backbone. eGFP - 3C - StrepII - 3xFlag Tag	Frances Diehl (*75*)

CRISPR Knockout Plasmids

Plasmid	Description	Source
pX330	pX330-U6-Chimeric_BB-CBh-hSpCas9	Addgene, 158973
pMC262	pX330 backbone. GCN2 sgRNA1, Ex9 nt1120	This Study

In vitro Transcription Oligos

Oligo	Description	Sequence	Source
prMC591	Leu CAG Template strand	TGGTGTCAGGAGTGGGATTCGAACCCACGCCTCCAGGGGAGACTGCGACCTGAACGCAGCGCCTTAGACCGCTCGGCCATCCTGACTATAGTGAGTCGTATTAAGCT	This Study
prMC595	T7 Fwd IVT primer 2	AGCTTAATACGACTCACTATAGG	This Study
prMC686	T7 Fwd IVT primer 3	AGCTTAATACGACTCACTATAG	This Study
prMC688	BsY_{CUA} Template Strand	TGGAGGGGGACGGATTCGAACCGCCGAACCCAAAGGGAGCGGATTTAGAGTCCGCCGCGTTTAGCCACTTCGCTACCCCTCCTATAGTGAGTCGTATTAAGCT	This Study
prMC591	Leu_{CAG} Template strand	TGGTGTCAGGAGTGGGATTCGAACCCACGCCTCCAGGGGAGACTGCGACCTGAACGCAGCGCCTTAGACCGCTCGGCCATCCTGACTATAGTGAGTCGTATTAAGCT	This Study
prMC733	Leu_{CAG} scrambled Template Strand	TGGTAGAGAGTACGCACGGCACCGTGTGCTACGCGATCGACGCGAGCCCTAAGTCCTTGCGCAACCTAGCACGCGTCCCGATGGGCTATAGTGAGTCGTATTAAGCT	This Study

bioRxiv preprint doi: https://doi.org/10.64898/2026.09.09.750423; this version posted September 13, 2026. The copyright holder for this preprint (which was not certified by peer review) is the author/funder, who has granted bioRxiv a license to display the preprint in perpetuity. It is made available under a CC-BY 4.0 International license.

Northern Probes

**Oligo**	**Description**	**Sequence**	**Source**
npMC4	BsY CUA Northern Probe 1	CGCCGCGTTTAGCCACTTCGCTACCCCTCC	This Study
npMC5	BsY CUA Northern Probe 2	AGGGAGCGGATTTAGAGTCCGCCGCGTTTA	This Study
npMC7	Gly_{GCC} Nortnern Probe	GCAGGCGAGAATTCTACCACTGAACCACCAATGC	Luisa Cochella Lab (*76*)

Antibodies

Primary Antibodies

Antibody	Dilution	Company	Catalog #
Rabbit Monoclonal α - Total GCN2	1:2000	Cell Signaling Technology	46041
Rabbit Monoclonal α - GCN2 (T899p)	1:1000	Cell Signaling Technology	94668
Mouse Monoclonal α - Total eIF2α	1:2000	Abcam	ab5369
Rabbit Monoclonal α - eIF2α (S52p)	1:1000	Abcam	ab32157
Rabbit Polyclonal α - uS3	1:5000	Abcam	ab140688
Rabbit Polyclonal α - eRF1	1:1000	Cell Signaling Technology	13916
Rabbit Monoclonal α - Tubulin β	1:2000	Cell Signaling Technology	2128
Rabbit Polyclonal α - GFP	1:5000	Abcam	ab6556
Mouse Monoclonal α - Vinculin	1:5000	Santa Cruz Biotechnology	sc-73614

Secondary Antibodies

Goat α - Rabbit IgG	1:5000	Cell Signaling Technology	7074
Horse α - Mouse IgG	1:5000	Cell Signaling Technology	7076

Protein Purification

GCN2

Human GCN2 was overexpressed in Expi293F (Thermofisher, A14527) by transfection of plasmid encoding wild-type hsGCN2 or mutants (see DNA Constructs). Cells were cultured in Expi293 Expression Medium (Thermofisher, A1435101). The day before transfection cells were subcultured to a density of 2.5-3E6 cells/ml in 25 ml. On the day of transfection cells were diluted

bioRxiv preprint doi: https://doi.org/10.64898/2026.09.09.750423; this version posted September 13, 2026. The copyright holder for this preprint (which was not certified by peer review) is the author/funder, who has granted bioRxiv a license to display the preprint in perpetuity. It is made available under a CC-BY 4.0 International license.

with fresh media to a density of 2.5E6 in 25 ml and transfected with 25 µg plasmid using Expifectamine293 (Thermofisher, A14524) according to the manufacturers protocol. 16 hours after transfection, ExpiFectamine™ 293 Transfection Enhancers 1 and 2 (Thermofisher, A14524) were added to the media, along with 2 µM GCN2 inhibitor A-92 (MedChemExpress, 1448693-69-3) and 500 nM ISRIB (Millipore Sigma, SML0843-5MG) to suppress activity of constitutively active mutants. Protein expression continued for 48 hours before cells were harvested by centrifugation at 300 x *g* for 5 min at 25°C and flash frozen in liquid nitrogen.

To purify the expressed protein, pellets were thawed on ice and resuspended in 25 ml of Buffer A (20 mM Tris·HCl pH 8.0, 150 mM NaCl, 5 % Glycerol, 2 mM β-mercaptoethanol (BME)). Resuspended pellets were supplemented with 1 tablet cOmplete EDTA-free protease inhibitor cocktail (Millipore Sigma, 4693159001), 500 U Benzonase Nuclease (Millipore Sigma, E8263-5KU), and 800 µl BioLock reagent (IBA LifeSciences, 2-0205-050). Cells were lysed by sonication using a probe sonicator with a ½" diameter horn (Branson Ultrasonics, SFX250) using a 1 min cycle at 50 % amplitude with 3 sec pulses followed by 9 second rests. The resulting lysate was clarified by centrifugation at 30,000 x *g* for 30 min at 4°C.

The clarified lysate was syringe filtered with a 0.45 µm SFCA filter (Corning, 431220) and loaded at 1 ml/min onto a 5 ml Strep-Tactin 4Flow high capacity FPLC column (IBA LifeSciences, 2-1258-001) equilibrated with 4 column volumes (C.V.) of Buffer A. The bound protein was washed with 10 C.V. of Buffer A before elution with 5 C.V. of Buffer A supplemented with 6 mM Desthiobiotin (IBA LifeSciences, 2-1000-002). The eluted protein was immediately loaded at 1 ml/mi onto a 5 ml HiTrap Q HP anion exchange column (Cytiva, 17115301) in equilibrated in Buffer A. The bound protein was washed with 5 C.V. of Buffer A at 2 ml/min flow and eluted with a 2 C.V. gradient from 100 % Buffer A to 100 % Buffer B (20 mM Tris·HCl pH 8.0, 1000 mM NaCl, 5 % Glycerol, 2 mM BME) under low flow, at 0.25 ml/min. The elution peak containing GCN2 was generally less than 1 ml and could be loaded directly onto a Superdex Increase S200 10/300 GL size exclusion chromatography column (Cytiva, 28990944) equilibrated in Buffer C (20 mM HEPES pH 7.5, 150 mM NaCl, 5 % Glycerol, 2 mM Tris(2-carboxyethyl)phosphine hydrochloride (TCEP)). Peak fractions were collected, diluted to 1 µM in Buffer C, and flash frozen in liquid nitrogen.

bioRxiv preprint doi: https://doi.org/10.64898/2026.09.09.750423; this version posted September 13, 2026. The copyright holder for this preprint (which was not certified by peer review) is the author/funder, who has granted bioRxiv a license to display the preprint in perpetuity. It is made available under a CC-BY 4.0 International license.

P-stalk

P-stalk heteropentamer was expressed in insect cells and purified based on published protocols (*29*). P-stalk bacmid encoding each of GST-TEV-uL10, RPLP1, and RPLP2 were generated by recombination between a donor plasmid (DNA constructs) and bacmid maintained in MAX Efficiency DH10Bac Competent Cells (Thermnofisher, 10361012) using the Bac-to-bac system (Thermofisher, 10359016) according to the manufacturer's protocols. Recombinant bacmid DNA was purified using PureLink HiPure miniprep kits (Thermofisher, K210002) and transfected into Expisf9 Cells (Thermofisher, A35243) grown to 2.5E6 cells/ml in 4 ml ExpiSf CD Medium (Thermofisher, A3767802) using Expifectamine Sf (Thermofisher, A38915) according to the manufacturer's protocol. Transfected cells were grown for 4-5 days until their viability dropped below 80 %. The resulting P0 baculovirus was harvested from the media by centrifugation at 500 x *g* for 5 min at 25 ^{o}C and stored at 4 ^{o}C. Virus was amplified by reinfecting 50 ml of cells grown to 2E6 cells/ml with 10 ul of P0 virus. Cell growth was monitored from 4-7 days and P1 virus was harvested as before when cell viability dropped below 80 %.

P-stalk proteins were expressed by infecting 50 ml of ExpiSf9 cells grown to 5E6 cells/ml with 1 ml of P1 24 hours after the addition of 1 ml of ExpiSf Enhancer (Thermofisher, A3767806). Expression continued for 72 hours before cells were harvested by centrifugation at 300 x *g* for 5 min and flash frozen in liquid nitrogen.

To purify the expressed protein, pellets were thawed on ice and resuspended in 50 ml of Buffer D (20 mM Tris·HCl pH 8.0, 100 mM NaCl, 5 % Glycerol, 5 mM Dithiothreitol (DTT)). Resuspended pellets were supplemented with 1 tablet cOmplete EDTA-free protease inhibitor cocktail (Millipore Sigma, 4693159001) and 500 U Benzonase Nuclease (Millipore Sigma, E8263-5KU). Cells were lysed by sonication using a probe sonicator with a ½" diameter horn using a 1 min cycle at 50 % amplitude with 3 sec pulses followed by 9 second rests. The resulting lysate was clarified by centrifugation at 30,000 x *g* for 30 min at 4°C.

The clarified lysate was syringe filtered and loaded onto a 5 ml GSTrap FF FPLC column (Cytiva, 17513101) equilibrated in 4 C.V. Buffer D. The bound protein was washed with 10 C.V. of Buffer D and incubated overnight with 5 ml of 30 µg/ml TEV protease diluted in 5 ml Buffer D on

bioRxiv preprint doi: https://doi.org/10.64898/2026.09.09.750423; this version posted September 13, 2026. The copyright holder for this preprint (which was not certified by peer review) is the author/funder, who has granted bioRxiv a license to display the preprint in perpetuity. It is made available under a CC-BY 4.0 International license.

the column. The next morning the cleaved protein was collected from the column and loaded at 1 ml/mi onto a 5 ml HiTrap Q HP anion exchange column in equilibriated in Buffer D. The bound protein was washed with 5 C.V. of Buffer D at 2 ml/min flow and eluted with a 10 C.V. gradient from 100 % Buffer D to 100 % Buffer E (20 mM Tris·HCl pH 8.0, 1000 mM NaCl, 5 % Glycerol, 5 mM DTT) at 1 ml/min. Peak fractions containing P-stalk proteins were concentrated to < 500 µl and loaded onto a Superdex Increase S200 10/300 GL size exclusion chromatography column equilibrated in Buffer F (20 mM HEPES, 50 mM KOAc, 5 mM $Mg(OAc)_2$, 5 % Glycerol, 2 mM TCEP). Peak fractions containing P-stalk proteins were collected, concentrated to 20 µM and flash frozen in liquid nitrogen.

eIF2α-NTD

eIF2α-NTD was expressed in bacterial cells and purified using on published protocols (*74*). BL21 *E. coli* (New England Biolabs, C2527H) were co-transformed with plasmids encoding eIF2α-NTD-His6 (DNA constructs) and pGTf2 (Takara, 3340), to express chaperones GroEL and Trigger Factor, and grown overnight at 37 ^{o}C in LB media supplemented with 100 ug/ml Carbenicillin and 25 ug/ml Chloramphenicol. Overnight cultures were diluted 1:100 into 1L of LB medium supplemented with the same antibiotics and grown at 37 ^{o}C. Chaperone expression was induced at OD 0.4 by addition of 10 ng/ml tetracycline. At OD 0.6, cultures were chilled to 20 ^{o}C, and eIF2α-NTD expression was induced by addition of 0.5 mM Isopropyl β-D-1-thiogalactopyranoside (IPTG, Gold Biotechnology, I2481C25). Expression continued for 6 hours at 20 ^{o}C before cells were harvested by centrifugation for 10 min at 4000 x *g* and flash frozen in liquid nitrogen.

The cell pellet from 2 L of culture was thawed on ice, resuspended in 50 ml of Buffer G (20 mM Tris·HCl pH 8.0, 300 mM NaCl, 25 mM Imidazole, 5 % Glycerol, 2 mM BME) supplemented with 1 tablet cOmplete EDTA-free protease inhibitor cocktail and 500 U Benzonase Nuclease, and lysed by sonication using a probe sonicator with a ½" diameter horn using a 2 minute cycle at 60 % amplitude with 3 sec pulses followed by 9 second rests. The resulting lysate was clarified by ultracentrifugation using a Ti45 rotor (Beckman Coulter, 339160) for at 40,000 rpm for 45 min at 4 ^{o}C.

bioRxiv preprint doi: https://doi.org/10.64898/2026.09.09.750423; this version posted September 13, 2026. The copyright holder for this preprint (which was not certified by peer review) is the author/funder, who has granted bioRxiv a license to display the preprint in perpetuity. It is made available under a CC-BY 4.0 International license.

The clarified supernatant was added to 1 ml of PureCube Ni-NTA agarose resin (Cube Biotech, 31103) equilibrated w/ 10 C.V. of Buffer G. The lysate was mixed with the resin for 1 h with gentle rotation at 4 °C and poured into a chromatography column. The unbound flow-through was removed and the beads were washed with 10 C.V, of Buffer G, followed by 10 C.V. of Buffer H (20 mM Tris·HCl pH 8.0, 1000 mM NaCl, 25 mM Imidazole, 5 % Glycerol, 2 mM BME) and a final 10 C.V. of Buffer G. Proteins bound after washing were eluted by 5 1 C.V. additions of Buffer I (20 mM Tris·HCl pH 8.0, 300 mM NaCl, 250 mM Imidazole, 5 % Glycerol, 2 mM BME), concentrated to < 1 ml, and loaded onto a Superdex 75 10/300 GL size exclusion column (Cytiva, 29148721) equilibrated in Buffer J (20 mM HEPES pH 7.5, 350 mM NaCl, 5 % Glycerol, 2 mM TCEP). Peak fractions were collected, concentrated to 100 μM, and flash frozen in liquid nitrogen.

Endogenous eIF2

Endogenous eIF2 trimer was purified from Expi293F cells based on published protocols (*77*, *78*). 400 ml of Expi293 cells were grown to a density of 5E6 cells/ml in Expi293 Expression Medium. The cells were pelleted by centrifugation at 2,500 x *g* for 15 min at 4 °C, washed in 40 ml ice cold 1X PBS (Thermofisher, 10010023), and pelleted again by centrifugation at 2,500 x *g* for 5 min at 4 °C. The washed pellet was resuspended in 25 ml Buffer K (10 mM HEPES pH 7.5, 10 mM KCl, 1.5 mM $MgCl_2$, 1 mM DTT, 1 X Halt Protease/Phosphatase Inhibitor Cocktail (Thermofisher, 78440)) and immediately pelleted by centrifugation at 2,500 x *g* for 5 min at 4°C. The pellet was resuspended in 15 ml ice cold Buffer K and incubated on ice for 10 min before being lysed by 10 strokes with the tight pestle of a 40 ml Kimble dounce homogenizer (Millipore Sigma, D9188-1SET). Unbroken nuclei were removed from the lysate by centrifugation at 3,000 x *g* for 20 min at 4 °C and the resulting supernatant was supplemented with 10 % Glycerol, 1 mM EDTA, and 1mM EGTA before clarification by centrifugation at 20,000 x *g* for 20 min at 4 °C. After clarification, the KCl concentration was adjusted to 450 mM and the lysate was ultracentrifuged at 45,000 rpm for 4 h at 4 °C in a Ti70 rotor (Beckman Coulter, 342184). The middle 2/3 of the supernatant was carefully extracted and stirred in a beaker at 4 °C for 1 h as saturated $(NH_4)_2SO_4$ was slowly added to a final concentration of 40 %. Precipitated proteins were removed by centrifugation at 20,000 x *g* for 10 min at 4 °C. The supernatant was again added to a beaker and stirred at 4 °C for 1 h as saturated $(NH_4)_2SO_4$ was slowly added to a final concentration of 50 %.

bioRxiv preprint doi: https://doi.org/10.64898/2026.09.09.750423; this version posted September 13, 2026. The copyright holder for this preprint (which was not certified by peer review) is the author/funder, who has granted bioRxiv a license to display the preprint in perpetuity. It is made available under a CC-BY 4.0 International license.

Precipitated proteins were again pelleted by centrifugation at 20,000 x *g* for 10 min at 4 ºC, and the pellet containing eIF2 was resuspended in 20 ml Buffer L (20 mM HEPES pH 7.5, 50 mM KCl, 10 % Glycerol, 1 mM DTT) and dialyzed overnight against 2 L of Buffer L.

The next morning, the protein was recovered and syringe filtered with a 0.45 µm SFCA filter before loading onto a monoQ 5/50 anion exchange column (equivalent to Capto HiRes Q 5/50, Cytiva, 29275878) equilibrated in Buffer L. Proteins washed with 5 C.V. of Buffer L and eluted in a 20 ml gradient from 100 % Buffer L to 100 % Buffer M (20 mM HEPES pH 7.5, 500 mM KCl, 10 % Glycerol, 1 mM DTT), and fractions containing eIF2 were pooled and dialyzed for 2 h against 2L of Buffer L. The protein was again syringe filtered and subjected to identical ion exchange chromatography protocol using a MonoS 5/50 cation exchange column (equivalent to Capto HiRes S 5/50, Cytiva, 29275877). Fractions containing eIF2 were pooled and concentrated to < 500 µl before loading onto a S200 increase size exclusion column equilibrated in Buffer N (20 mM HEPES pH 7.5, 150 mM KCl, 10 % Glycerol, 1 mM DTT). Peak fractions containing eIF2 were pooled, concentrated to 2.5 µM, and flash frozen in liquid nitrogen.

RNA Purifications

Ribosomes

80S ribosomes were purified from rabbit reticulocyte lysate (RRL, Green Hectares). 6 ml of crude RRL was split 4 ways, layered 1.5 ml per tube over 1 ml sucrose cushions made of Buffer O (50 mM Tris·HCl pH 8.0, 500 mM KOAc, 25 mM $Mg(OAc)_2$, 1 mm DTT, 500 mM Sucrose) in 3.5 ml open-top polycarbonate centrifuge tubes (Beckman Coulter, 349622), and centrifuged at 100,000 x *g* for 1 h at 4 ºC in a TLA100.3 rotor (Beckman Coulter, 349481). The resulting pellets were resuspended in 1.5 ml total volume of Buffer P (50 mM HEPES pH 7.5, 500 mM KCl, 2 mM $MgCl_2$, 2 mM DTT). To separate the ribosomal subunits, 2 mM Puromycin Dihydrochloride (Millipore Sigma, P7255-25MG) was added to the resuspension, and the ribosomes were incubated sequentially on ice for 15 min, at 37 ºC for 10 min with gentle agitation, and on ice for 10 min. This mixture was then layered, 250 µl per tube, above 12 ml gradients from 10 – 35 % sucrose made in Buffer P with a Biocomp Gradient Master (Biocomp Instruments, Model 108).

bioRxiv preprint doi: https://doi.org/10.64898/2026.09.09.750423; this version posted September 13, 2026. The copyright holder for this preprint (which was not certified by peer review) is the author/funder, who has granted bioRxiv a license to display the preprint in perpetuity. It is made available under a CC-BY 4.0 International license.

The gradients were centrifuged at 40,000 rpm for 1:45 at 4 °C in open-top 12 ml PolyClear tubes (Seton Scientific, 7030) using a Sw41 Ti swinging bucket rotor (Beckman Coulter, 331362). The gradients were fractionated into 20 500 µl fractions using a piston fractionator (Biocomp Instruments, Model 152) with continual $Absorbance_{260}$ monitoring using a Triax flow cell (Science Services, FC-2). Fractions containing 40S and 60S subunits were pooled and dialyzed overnight against 2 L Buffer Q (20 mM HEPES pH 7.5, 100 mM KOAc, 5 mM $Mg(OAc)_2$, 2 mM DTT).

The next day, the dialyzed ribosomes were concentrated to < 1.5 ml using a 15 ml 100 kDa centrifugal concentrator (Millipore Sigma, UFC910008). The resulting concentrate was layered, 250 µl per tube, above 12 ml gradients from 10 – 35 % sucrose made in Buffer Q with a Biocomp Gradient Master. The gradients were centrifuged at 40,000 rpm for 1:45 at 4 °C in open top 12 ml PolyClear tubes using a Sw41 Ti swinging bucket rotor. The gradients were fractionated into 20 500 µl fractions using a piston fractionator. Fractions containing 80S monosomes were pooled and dialyzed overnight against 2 L of Buffer Q. The next day the ribosomes were concentrated to 2 µM using a 15 ml 100 kDa centrifugal concentrator and flash frozen in liquid nitrogen.

Bulk tRNA

Total tRNA was purified from pig liver. 600 g pig liver (Pel-Freez, 59423-2) was cut into pieces and homogenized in 1 L ice-cold Buffer R (20 mM Tris·HCl pH 7.5, 100 mM KCl) using a Waring blender (Waring Laboratory Science, 700S). The homogenate was centrifuged at 9,500 rpm for 16 min at 4 °C using a FiberLite F9-6 x 1000 Rotor (Thermofisher, 096-061075) with 1 L PPCO bottles (Thermofisher, 010-1491) resistant to 50 % phenol. The resulting supernatant was mixed with an equal volume phenol (Millipore Sigma, P9346) and stirred at room temperature for 30 min. The mixture was again centrifuged at 12,000 rpm for 15 min at room temperature to separate the phases. The aqueous phase was collected in a new flask and stirred with equal volume chloroform (Millipore Sigma, C2432-1L) for 30 min at room temperature. The mixture was again centrifuged at 12,000 rpm for 15 min. The resulting aqueous phase was collected, mixed with 0.1 volume of 3M NaOAc pH 5.0 and 3 volumes of 95 % ethanol, and stored at -20 °C overnight.

bioRxiv preprint doi: https://doi.org/10.64898/2026.09.09.750423; this version posted September 13, 2026. The copyright holder for this preprint (which was not certified by peer review) is the author/funder, who has granted bioRxiv a license to display the preprint in perpetuity. It is made available under a CC-BY 4.0 International license.

The next morning, the precipitate was centrifuged at 8, 000 rpm for 20 min at 4°C and the resulting crude RNA pellet was washed with 70 % ethanol, air dried and resuspended in 200 ml Buffer R and centrifuged one final time at 8, 000 rpm for 20 min at 4°C to remove any insoluble contaminants. The crude RNA was then loaded onto a 17 ml column packed DEAE Sepharose fast flow resin (Cytiva, 17070960) equilibrated in Buffer R. The column was washed with > 200 ml of Buffer R until the A_{260} of the flow-through dropped below 0.3. The bound tRNAs were eluted in 200 ml Buffer S (20 mM Tris·HCl pH 7.5, 100 mM KCl, 1 M NaCl), mixed with 400 ml 95 % ethanol, and stored at -20 °C overnight. The following morning the precipitate was centrifuged at 8,000 rpm for 25 min at 4 °C. The pellet was washed with 70 % ethanol, air dried, resuspended in Ambion Nuclease Free water (Thermofisher, AM9932) at 10 mg/ml, and flash frozen in liquid nitrogen.

These total tRNA were considered partially acylated and were chemically deacylated by alkaline hydrolysis. 10 mg of total tRNA were thawed on ice, mixed with 0.1 volume of 3M NaOAc pH 5.0 and 3 volumes of 95 % ethanol, and incubated at -20 °C for 1 h. The resulting precipitate was centrifuged at 20,000 x g for 30 min at 4 °C, washed twice with 70 % ethanol, and air dried. The pellet was resuspended in 950 ul nuclease free water, and the pH raised by addition of 50 µl of 1 M Tris·HCl pH 9.0. The tRNA were incubated at pH 9.0 for 1 h at 37 °C and then purified by a second ethanol precipitation. The final pellet was resuspended at 10 mg/m in nuclease-free water and flash frozen with liquid nitrogen. These tRNAs were considered deacylated throughout this study.

In vitro transcribed tRNAs

tRNAs and scrambled RNAs were transcribed with the MegaShortScript T7 transcription kit (Thermofisher, AM1354) according to the manufacturers protocol. Single-stranded template DNA oligos including both the T7 promoter and tDNA sequence (DNA constructs) were annealed with ssDNA oligos encoding the T7 complement sequence at 2.5 µM final concentration in Buffer T (10 mM Tris·HCl pH 8.0, 100 mM NaCl, 1 mM EDTA). The oligos were fully denatured by heating to 95 °C for 3 min and then annealed by gradual cooling to room temperature. 50 µl reactions

bioRxiv preprint doi: https://doi.org/10.64898/2026.09.09.750423; this version posted September 13, 2026. The copyright holder for this preprint (which was not certified by peer review) is the author/funder, who has granted bioRxiv a license to display the preprint in perpetuity. It is made available under a CC-BY 4.0 International license.

were assembled from kit components with 250 nM annealed oligos, 7.5 mM each of ATP, CTP, GTP, UTP, 1 X T7 Reaction Buffer, and 5 µl T7 Enzyme Mix. Reactions were incubated at 37 °C overnight. The next morning, the DNA template was degraded by addition of 5 U TURBO DNase (Thermofisher, AM2238) at 37 °C for 15 min. Transcription products were purified using RNA Clean and Concentrate columns (Zymo Research, R1019), eluted in 200 µl nuclease-free water, and flash frozen in liquid nitrogen at 1 mg/ml.

Cell Culture

HEK293T cells (ATCC, CRL-3216) were cultured in Dulbecco's Modified Eagle Medium (DMEM; Thermofisher, 11995065) supplemented with 10 % fetal bovine serum (FBS; Thermofisher, 26140079) in 10 cm cell culture dishes (Corning, 430167) at 37 °C in a 5 % CO_2 humidified incubator and passaged every 2-3 days. HEK293T cells were thawed and passaged at least twice before their use in experiments. GCN2 KO cells were produced from parental HEK293T cells by transfection of pMC262 (DNA constructs). Cells were grown for 48 hours post-transfection before dilution with fresh media to single cells in 96-well plates (USA Scientific, CC7682-7596). Cells were outgrown for 14 days and colonies validated as GCN2 knockouts by western blotting and PCR. Positive colonies were selected for clonal expansion and cryogenically stored. PKR KO cells, a gift from Chien-I Yang, were similarly generated from parental HEK293T cells.

Transfection

tRNA transfections in Figure 3 were performed in PKR KO cells. GCN2 transfections in Figure 5 were performed in GCN2 KO cells. Cells were seeded at 2E6 cells/plate 24 hours prior to transfection and supplied with fresh media 1 hour before transfection. Cells were transfected with the indicated plasmids (DNA Constructs) using Lipofectamine 3000 (Thermofisher, L3000001) according to the manufacturer's protocol. 5 µg plasmid DNA was transfected per 10 cm dish. No

bioRxiv preprint doi: https://doi.org/10.64898/2026.09.09.750423; this version posted September 13, 2026. The copyright holder for this preprint (which was not certified by peer review) is the author/funder, who has granted bioRxiv a license to display the preprint in perpetuity. It is made available under a CC-BY 4.0 International license.

DNA transfection controls were processed identically, including transection reagents but omitting DNA.

Cellular Treatment and Lysis

For Histidine starvation experiments, DMEM was removed by gentle aspiration 16 – 24 hours after transfection and the cells were washed once with 1X PBS before introduction of DMEM -Histidine Media (USBiologicals, D9801-02). After 1 h, the medium was removed by gentle aspiration and cells were washed rapidly in 1X PBS and harvested by scraping in 250 µl Buffer U (50 mM HEPES pH 7.4, 100 mM KOAc, 15 mM $Mg(OAc)_2$, 5 % Glycerol, 0.5 % Triton X-100 (Millipore Sigma, X100-100ML)) supplemented with 1X Halt Protease/Phosphatase Inhibitor Cocktail, 80 U TURBO DNase. Lysates were clarified by centrifugation at 8,000 x *g* for 5 min at 4°C and either flash frozen in liquid nitrogen or directly used for western blotting or sucrose gradient sedimentation.

SRI-41315 experiments were performed in a similar manner. 16-24 hours after transfection, the cells were supplied with fresh media. After an hour, SRI-41315 (Millipore Sigma, SML3312-5MG) was added to a final concentration of 3.3 or 10 µM, as indicated. Control cells were treated with an equal volume of DMSO (Thermofisher, D12345). Cells were cultured for the indicated time in the presence of the drug before being washed rapidly in 1X PBS and harvested by scraping in 250 µl Buffer U (50m M HEPES pH 7.4, 100mM KOAc, 15mM $Mg(OAc)_2$, 5 % Glycerol, 0.5 % Triton X-100 (Millipore Sigma, X100-100ML), 1 X Halt Protease/Phosphatase Inhibitor Cocktail, 80 U TURBO DNase. Lysates were clarified by centrifugation at 8,000 x *g* for 5 min at 4°C and either flash frozen in liquid nitrogen or directly used for western blotting or sucrose gradient sedimentation.

Kinase Assay

Proteins and RNAs were thawed on ice and diluted to 10 X assay concentrations in Buffer V (20 mM HEPES pH 7.4, 50 mM KOAc, 5 mM $Mg(OAc)_2$. Reactions were assembled in Buffer V

bioRxiv preprint doi: https://doi.org/10.64898/2026.09.09.750423; this version posted September 13, 2026. The copyright holder for this preprint (which was not certified by peer review) is the author/funder, who has granted bioRxiv a license to display the preprint in perpetuity. It is made available under a CC-BY 4.0 International license.

with 0.5 mM TCEP, and 0.5 mg/ml recombinant albumin (NEB, B9200S), and the indicated proteins, RNAs, polyinosine-polycytidylic acid (polyIC; InvivoGen, tlrl-picw), or polyadenylic acid (polyA; Millipore Sigma, 10108626001). Typical reaction conditions included 20 nM GCN2, 2 µM eIF2α-NTD, 200 nM P-stalk, and 5 µM tRNA, though many variations were performed as outlined in the figures. Single timepoint reactions were assembled to a final volume of 9 µl/reaction. Reactions for kinetic experiments were mixed at the same proportions such that 10 µl aliquots could be removed at each time point, with 10 µl remaining unused at the end. Once assembled, reactions were moved to 17 ^{o}C and incubated without ATP for 15 min. After this pre-incubation, the reaction was supplemented 500 µM (final concentration) ATP and 0.25 µCi/µl γ^{32}P-labeled ATP (Revvity, BLU502A001MC), bringing the final volume to 10 µl/reaction, and returned to 17 ^{o}C. At the indicated time points, 9 µl reaction was removed and quenched with 3 µl 4 X Laemmli Buffer (200 mM Tris·HCl pH 6.8, 8 % Sodium Dodecyl Sulfate, 0.4 % Bromophenol blue, 40 % Glycerol).

Immunoprecipitations

Proteins and RNAs were thawed on ice. 50 µl reactions were assembled with the indicated proteins and RNAs in Buffer V with 0.5 mM TCEP, 0.5 mg/ml recombinant albumin, and 0.1 % Tween-20 (Millipore Sigma, P1379-25ML). Typical reactions contained 200 nM GCN2, 2 µM eIF2α-NTD, 2 µM P-stalk, 0 – 5 µM tRNA, though other variations were tested, as indicated. Reactions were incubated at 17 ^{o}C for 15 min, and then 40 µl was added to 0.2 µl MagStrep Strep-Tactin XT beads (IBA Lifesciences, 2-5090-010) equilibrated in Buffer V supplemented with 0.1 % Tween-20, with the rest reserved as an input sample. Reactions were incubated with beads for 1 hour at room temperature and then the unbound fraction was removed using a magnetic tube rack (Zymo Research, 3DP-1002). Beads were washed 3 times with Buffer V supplemented with 0.5 mM TCEP, 0.05 mg/ml recombinant albumin, and 0.1 % Tween-20 and three times with Buffer W (100 mM Tris·HCl pH 8.0, 200 mM NaCl, 1 mM EDTA, 0.1 % Tween-20) before elution in 20 µl Laemmli Buffer or by sequential 15 min incubations with 10 µl Buffer Y (100 mM Tris·HCl pH 8.0, 150 mM NaCl, 1 mM EDTA, 50 mM biotin (IBA Lifesciences, 2-1016-005)).

bioRxiv preprint doi: https://doi.org/10.64898/2026.09.09.750423; this version posted September 13, 2026. The copyright holder for this preprint (which was not certified by peer review) is the author/funder, who has granted bioRxiv a license to display the preprint in perpetuity. It is made available under a CC-BY 4.0 International license.

Sucrose Gradient Sedimentation

Sucrose gradients were prepared from stocks of 10 % and 50 % sucrose in Buffer Z (25 mM HEPES pH 7.5, 100 mM KOAc, 5 mM $Mg(OAc)_2$, 1 mM TCEP) using a Biocomp Gradient Master and chilled at 4 °C overnight. Samples from cellular lysates were normalized by total protein with Pierce BCA assays (Thermofisher, 23225), and 200 µl lysate, ~ 1mg of total protein, was loaded onto each gradient. Gradients were centrifuged at 40,000 rpm for 1:45 at 4 °C in open-top 12 ml PolyClear tubes using a Sw41 Ti swinging bucket rotor. Gradients were fractionated with a Biocomp piston fractionator and A_{260} profiles of the gradients were recorded with a Triax flow cell.

Coomassie Staining and Autoradiography

Reactions to be analyzed by Coomassie stain and autoradiography were separated by gel electrophoresis using 4-20 % Tris Glycine TGX gels (Biorad, 5671095) run in 1 X Tris Glycine Running Buffer (Biorad, 1610732) at 150 V for 60 min. Afterwards, gels were fixed in Buffer X (40 % methanol, 10 % acetic acid), stained for 15 min in Coomassie R250 stain (0.1 % Coomassie R-250 (Thermofisher, 20278), 40 % methanol, 10 % acetic acid), and destained for 2 hours in Buffer V. Destained gels were imaged on a GelDoc Imager (Biorad, 12009077) before drying on a vacuum gel dryer (Biorad, Model 583) for 1 hour at 70 °C. Dried gels were exposed to magnetic phosphor screens (Cytiva, 28956476) for 30 min (substrate phosphorylation) or overnight (autophosphorylation) and imaged with a Typhoon Imager (Cytiva, 29187193) using 100 µM pixel size and 1000 V PMT.

Immunoblotting

Samples from cellular lysates were normalized by total protein with Pierce BCA assays and 10 µg total protein was loaded in each gel lane. Kinase reactions were diluted 1:10 and 8 µl was loaded in each lane. Electrophoresis was performed as described in "Autoradiography." Proteins

bioRxiv preprint doi: https://doi.org/10.64898/2026.09.09.750423; this version posted September 13, 2026. The copyright holder for this preprint (which was not certified by peer review) is the author/funder, who has granted bioRxiv a license to display the preprint in perpetuity. It is made available under a CC-BY 4.0 International license.

were transferred to 0.2 µm Immun-Blot PVDF membranes (Biorad, 1620175) in 1X Trans-Blot Turbo Buffer (Biorad, 10026938) using a Trans-Blot Turbo Semi-Dry transfer system (Biorad, 1704150) with a 10 min, 2.5 A protocol.

Membranes were blocked with 5 % non-fat milk solids (Santa Cruz Biotechnology, sc-2324) in 1X TBST (20 mM Tris·HCl pH 7.5, 150 mM NaCl, 0.1 % Tween-20) for 1 hour. Primary antibodies were diluted in 5 % non-fat milk solids in 1X TBST and incubated with membranes overnight at 4 ^{o}C with gentle rocking. The next morning membranes were washed 4 times for 5 min in 1X TBST before addition of HRP-conjugated secondary antibodies diluted in 5 % non-fat milk solids in 1X TBST. Membranes were incubated with secondary antibodies for 1 hour at room temperature and then washed 4 times with 1X TBST for 5 min. Membranes were then dried, exposed for 2 min to SuperSignal Pico PLUS (Thermofisher, 34580) or Femto Maximum Sensitivity (Thermofisher, 34094) substrate, and imaged by chemiluminescence with a GelDoc Imager.

Northern Blotting

Total RNA was extracted from cellular lysates by mixing 100 µl lysate with 1 ml TRIzol reagent (Thermofisher, 15596026). Samples were vortexed briefly and incubated 5 min at RT. 200 µl chloroform (Millipore Sigma, C2432) was added and the samples were mixed thoroughly and centrifuged at 12,000 x *g* for 15 min at 4 ^{o}C. The resulting aqueous phase was mixed with 500 µl ice-cold isopropanol, incubated for 10 min at 4 ^{o}C, and centrifuged at 12,000 x *g* for 10 min at 4 ^{o}C. The precipitated RNA pellet was washed twice with 75 % ethanol, air dried, and resuspended in 20 µl TE buffer (Thermofisher, AM9849). RNA concentration was measured by Qubit Broad Range (BR) assay (Thermofisher, Q10210) and 10 µg was mixed with equal volume 2X RNA loading dye (NEB, B0363S), heated at 95 ^{o}C for 3 min, cooled on ice, and loaded on a 10 % TBE-Urea PAGE gel (Biorad, 3450089). Gels were run at 200 V for 90 min in 1X TBE buffer (Thermofisher, 15581044) and transferred to a Hybond-N+ membrane (Cytiva, 15581044) in 1X TBE using a TransBlot Turbo Semi-Dry transfer system in 1X TBE with a 30 min 1.0 A protocol. Membranes were crosslinked using a Stratalinker 2400 UV crosslinker (Agilent Technologies, 400075) using the “Auto” setting and then washed twice with 2X SSC buffer (Thermofisher, AM9763).

bioRxiv preprint doi: https://doi.org/10.64898/2026.09.09.750423; this version posted September 13, 2026. The copyright holder for this preprint (which was not certified by peer review) is the author/funder, who has granted bioRxiv a license to display the preprint in perpetuity. It is made available under a CC-BY 4.0 International license.

Prior to hybridization, membranes were incubated in NorthernMax hybridization buffer (Thermofisher, AM8677) at 42 °C for 60 min with gentle rotation. 50 pmol probe was labeled with 50 pmol of γ^{32}P-labeled ATP (Revvity, BLU035C005MC) using T4 polynucleotide kinase (NEB, M0201S) in 50 µl reactions. Excess nucleotides were removed with MicroSpin G-50 columns (Cytiva, 27533001). 10 µl Phosphorylated probe was diluted in 25 ml pre-warmed hybridization buffer and incubated with membranes at 42 °C overnight with gentle rotation. The next morning the membranes were washed 3 times for 20 min with 2X SSC for 20 before exposure to a magnetic phosphor screen and imaging by autoradiography with a Typhoon imager.

Data Analysis

Autoradiographs and Coomassie stained images were analyzed using ImageJ (https://imagej.net/ij/). ROIs were contrast adjusted using the Adjust Brightness and Contrast "Auto" setting and background corrected with a 50 pixel rolling ball radius. Band intensities were extracted using the built in "Analyze Gel" toolkit. The resulting values were normalized in Matlab (www.mathworks.com/). For Coomassie stained gels, the integrated intensity of the upper, phosphorylated, eIF2α-NTD band was divided by the sum of the upper and lower bands to yield the fraction phosphorylated. For each kinetic experiment, the fraction phosphorylated measured by Coomassie stain at the longest timepoint was used to normalize the integrated intensity of the autoradiographic signals of substrate phosphorylation to produce the fraction phosphorylated values reported. The autoradiographic signals for autophosphorylation lack a Coomassie stain normalization and were instead normalized to the highest single band intensity on the gel to produce a relative phosphorylation. The fractional phosphorylations from substrate phosphorylation time courses were plotted, along with standard error of the mean (SEM) at each timepoint, in GraphPad Prism (https://www.graphpad.com/) and fit with a two-term hypo-exponential model:

$$Y = 1 - e^{-j*X} * \frac{k}{k-j} - e^{-k*X} * \frac{j}{j-k}$$

bioRxiv preprint doi: https://doi.org/10.64898/2026.09.09.750423; this version posted September 13, 2026. The copyright holder for this preprint (which was not certified by peer review) is the author/funder, who has granted bioRxiv a license to display the preprint in perpetuity. It is made available under a CC-BY 4.0 International license.

The relative phosphorylations from autophosphorylation were plotted in Graphpad, along with SEM at each timepoint and fit with the software's default exponential association model. Fits were differentiated with the GraphPad "Analysis, Differentiate" function with no smoothing, and the maximum of the derivative was plotted as a function of concentration to establish the dosage response of agonism or antagonism. These curves were fit with GraphPad's 3 or 4 parameter dosage responses curves or the Michaelis-Menten equation using default settings to yield estimates of EC_{50} or IC_{50}.

Structural Modeling

Structural predictions were generated with Alphafold3 (*79*) in the Google Collab environment (https://alphafoldserver.com/) using default settings. The residues and nucleotides included in each model are indicated in the figure legends. Structural predictions were visualized in UCSF ChimeraX (https://www.cgl.ucsf.edu/chimerax/).

Sequence Alignment

GCN2 protein sequences from all *Chordata* species annotated were downloaded from the NCBI RefSeq database (https://www.ncbi.nlm.nih.gov/refseq/). Sequences were limited to one, longest, isoform per species. Sequences were aligned using Clustal Omega (*80*) in JalView (https://www.jalview.org/).

bioRxiv preprint doi: https://doi.org/10.64898/2026.09.09.750423; this version posted September 13, 2026. The copyright holder for this preprint (which was not certified by peer review) is the author/funder, who has granted bioRxiv a license to display the preprint in perpetuity. It is made available under a CC-BY 4.0 International license.

Supplementary Figure Captions:

Figure S1. (A) Coomassie stained gel with 1-2 pmol of recombinant GCN2 variants used in this study. **(B)** Coomassie stained gel with 35 pmol purified P-stalk, 10 pmol eIF2 trimer, and 75 pmol of all eIF2α-NTD variants used in this study. **(C)** Schematic of a kinase assay using recombinant GCN2 (green), 80S ribosomes (gray), and eIF2α-NTD (light blue). **(D)** Coomassie stain, autoradiographs, and immunoblots with the indicated antibodies of kinase assays as in (B) in the presence or absence of 20 nM 80S ribosomes with 10 nM wild-type (WT) or kinase-dead D848N GCN2 and 2 µM eIF2α-NTD. **(E)** Intensities of autophosphorylation and substrate phosphorylation autoradiographs at the indicated P-stalk concentrations as in (Figure 1D) plotted and fit with single or hypo-exponential functions, respectively. Error bars represent S.E.M. of 4 independent replicates. **(F)** Coomassie stain, autoradiograph, and immunoblot with the indicated antibodies of kinase assays as in (Figure 1A) in the presence or absence of 200 nM P-stalk with 10 nM of the indicated GCN2 variants and 2 µM eIF2α-NTD.

Figure S2. (A) Coomassie stain, autoradiographs, and immunoblots with the indicated antibodies of kinetic kinase assays with 1000 nM P-stalk and a titration of tRNA as outlined in (Figure 2D). **(B)** Coomassie stain, autoradiographs, and immunoblots with the indicated antibodies of kinetic kinase assays with 200 nM P-stalk and a titration of tRNA as outlined in (Figure 2D). **(C)** Coomassie stain and autoradiographs of kinase assays with 20 nM GCN2, 200 nM P-stalk, 2 µM eIF2α-NTD, and a titration of purified, deacylated pig liver tRNA or *in vitro* transcribed Leu_{CAG} tRNA. **(D)** Coomassie stain and autoradiographs of kinase assays with 20 nM GCN2, 200 nM P-stalk, 500 nM purified endogenous eIF2 trimer, and 5 µM tRNA as indicated. **(E)** Coomassie stain and autoradiographs of kinase assays with 20 nM GST-PERK kinase domain (KD), 2 µM of eIF2α-NTD, and a titration of tRNA.

Figure S3. (A) Coomassie stain and autoradiographs of kinase assays with 20 nM GCN2, 200 nM P-stalk, 2 µM eIF2α-NTD, and a titration of purified, deacylated pig liver tRNA or *in vitro* transcribed BsY_{CUA} amber suppression tRNA. **(B)** Northern blots with the indicated probes of

bioRxiv preprint doi: https://doi.org/10.64898/2026.09.09.750423; this version posted September 13, 2026. The copyright holder for this preprint (which was not certified by peer review) is the author/funder, who has granted bioRxiv a license to display the preprint in perpetuity. It is made available under a CC-BY 4.0 International license.

BsY_{CUA} and Gly_{GCC} expression in cells transfected with no DNA, GFP (pFD042), or BsY amber suppression plasmids (pIRE4-Azi) in the presence or absence of Azido-Phenylalanine (AzF). **(C)** Immunoblots of GCN2 activity in HEK293T cells treated with 0, 3.3, and 10 µM SRI-41315 for 4 h to induce degradation of eRF1. **(D)** Immunoblots of GCN2 activity in wild-type (WT) and GCN2 KO HEK293T cells treated with 10 µM SRI-41315 for the indicated time course to induce degradation of eRF1.

Figure S4. (A) Coomassie stain and immunoblots with the indicated antibodies of kinase assays with 20 nM wild-type (WT) or F1143L/R1144L (m2) GCN2, 2 µM eIF2α-NTD, and the indicated titration of P-stalk. **(B)** Coomassie stains of co-immunoprecipitation experiments with 200 nM Strep-tagged GCN2 and 1 µM P-stalk in the presence of a titration of tRNA. The region including uL10 is displayed at two separate contrast levels for clarity. **(C)** Structural models of *Thermus thermophilus* Histidine tRNA Synthetase (SyH; residues 2-421, *tt*SyH; gray) with $tRNA_{His}$ (*tt*tRNA-His-GTG-1-1; yellow) (Crystal structure, PDB 4RDX (*81*)), *Homo sapiens* Histidine tRNA Synthetase (HARS, residues 1-509; *hs*HARS1, gray) with $tRNA_{His}$ (*hs*tRNA-His-GTG-1-1; yellow) (Alphafold3, top 5 predictions), *Homo sapiens* GCN2 Histidine tRNA Synthetase-like domain (HisRS; residues 1022-1493, *hs*EIF2AK4; green) with $tRNA_{His}$ (*hs*tRNA-His-GTG-1-1; yellow) (Alphafold3, top 5 predictions), and *Homo sapiens* GCN2 HisRS (HisRS; residues 1022-1493, *hs*EIF2AK4; green) with the C-terminal tail of P-stalk protein 2 (RPLP2; residues 106-115, *hs*RLA2; pink) (Alphafold3, top 5 predictions). The 5' and 3' ends of the tRNAs are colored orange and blue, respectively. The motif 2 (m2) loop of the GCN2 HisRS domain is colored red. **(D)** Coomassie stain and immunoblot with the indicated antibody of kinase assays with 20 nM of the indicated GCN2 variants and 2 µM eIF2α-NTD at the indicated time points. **(E)** As in (D), but at 60 min only with the indicated titration of tRNA. **(F)** Coomassie stain as in (D) with the indicated GCN2 variants. **(G)** As in (E) but at 60 min only with the indicated titration of tRNA. **(H)** Coomassie stain and autoradiographs of kinase assays with 20 nM GCN2, 200 nM P-stalk, 2 µM eIF2α-NTD, and a titration of partially acylated and deacylated pig liver tRNA. **(I)** As in (H) but with titrations of *in vitro* transcribed Leu_{CAG} or scrambled tRNAs. **(J)** Coomassie stain and immunoblots with the indicated antibodies of kinase assays as in (H), but with titrations of equivalent masses of purified tRNA, polyIC, and polyA.

bioRxiv preprint doi: https://doi.org/10.64898/2026.09.09.750423; this version posted September 13, 2026. The copyright holder for this preprint (which was not certified by peer review) is the author/funder, who has granted bioRxiv a license to display the preprint in perpetuity. It is made available under a CC-BY 4.0 International license.

Figure S5. (A) Coomassie stain and autoradiographs of kinetic kinase assays with 200 nM P-stalk and a titration of tRNA as outlined in (Figure 4B). **(B)** Coomassie stain, autoradiographs, and immunoblots with the indicated antibodies of kinetic kinase assays with 1000 nM P-stalk and a titration of eIF2α-NTD as outlined in (Figure 4G). **(C)** Intensities of substrate autoradiographs as in (B) plotted and fit with hypo-exponential functions. Error bars represent S.E.M. of 2 independent replicates. **(D)** V_0 in (C) as a function eIF2α-NTD concentration fit with the Michaelis-Menten equation (see Methods). Error bars represent S.E.M. of 2 different replicates. Error on reported K_m is the standard error of the fit. **(E)** Coomassie stain, autoradiographs, and immunoblots with the indicated antibodies of 30 min kinase assays with 20 nM GCN2 in the presence or absence of 200 nM P-stalk and 500 nM purified endogenous eIF2 trimer. **(F)** Coomassie stains from co-immunoprecipitation experiments with 200 nM Strep-tagged GCN2 and 2 µM eIF2α-NTD. The region including eIF2α-NTD is displayed at two separate contrast levels for clarity.

Figure S6. (A) Structural models of *Homo sapiens* PKR kinase domain (PKR(KD); residues 258-541, *hs*EIF2AK2, grey) with *Saccharomyces cerevisiae* eIF2α-NTD (eIF2α-NTD; residues 5-176, *sc*SUI2; light blue) (Crystal structure, PDB 2A1A (*38*)), *Homo sapiens* GCN2 kinase domain (GCN2(KD); residues 585-1016, *hs*EIF2AK4; green) with *Homo sapiens* eIF2α-NTD (eIF2α-NTD; residues 2-185, *hs*IF2A; light blue) (Alphafold3), *Homo sapiens* GCN2 kinase domain (GCN2(KD); residues 584-1006, *hs*EIF2AK4; green) (Crystal structure, PDB 7QQ6(*15*)). The αG helices of the kinase domains are colored in darker shades, with the Threonine residues corresponding to T943 in red. The activation loops of the kinases are colored yellow. **(B)** Clustal Omega alignments of the activation segments of the human ISR kinases. The disorded activation loop is indicated in dark font. The phosphorylated Threonine residues are colored blue. **(C)** Coomassie stains and autoradiographs from kinetic kinase assays with 20 nM of chimeric GCN2 constructs whose activation loop has been replaced by that of HRI, PKR, or PERK, with 200 nM P-stalk, in the presence or absence of 2 µM eIF2α-NTD. Autoradiographs are displayed at two separate contrast levels for clarity. **(D)**

bioRxiv preprint doi: https://doi.org/10.64898/2026.09.09.750423; this version posted September 13, 2026. The copyright holder for this preprint (which was not certified by peer review) is the author/funder, who has granted bioRxiv a license to display the preprint in perpetuity. It is made available under a CC-BY 4.0 International license.

Coomassie stain, autoradiographs, and immunoblots with the indicated antibodies of 10 min kinase assays with 20 nM of the indicated GCN2 variants and 200 nM P-stalk in the presence or absence of 2 µM eIF2α-NTD. **(E)** Coomassie stains from co-immunoprecipitation experiments with 200 nM Strep-tagged GCN2 variants and 2 µM eIF2α-NTD. The region including eIF2α-NTD is displayed at two separate contrast levels for clarity.

Figure S7. (A) Alphafold3 structural models of *Homo sapiens* Aurora Kinase B kinase domain (AURKB; residues 77-327, *hs*AURKB), *Homo sapiens* HRI kinase domain (HRI; residues 167-583. *hs*EIF2AK1), *Homo sapiens* PKR kinase domain (PKR; residues 267-538, *hs*EIF2AK2), *Homo* sapiens PERK kinase domain (PERK; residues 593-1077, *hs*EIF2AK3), Homo *sapiens* GCN2 kinase domains (GCN2; residues 585-1016, *hs*EIF2AK4). The kinase domain insertion loop is colored in magenta. **(B)** Clustal Omega alignment of the insertion loops of the kinases in (A). The insertion loop is indicated by darker font. S/T residues are colored in cyan. D/E residues are colored in magenta.

Figure S8. (A) Coomassie stain and autoradiographs of kinetic kinase assays with 10 nM wild-type (WT) or kinase insertion loop deletion (ΔKDloop) GCN2 and 200 nM P-stalk in the presence or absence of 2 µM eIF2α-NTD. **(B)** Coomassie stain and autoradiographs of 30 min kinase assays with 20 nM wild-type (WT) or kinase insertion loop deletion (ΔKDloop) GCN2, 500 nM P-stalk, 2 µM eIF2α-NTD, and the indicated tRNA titration. **(C)** Coomassie stain and autoradiographs of 30 min kinase assays with the indicated GCN2 variants and 2 µM eIF2α-NTD in the presence or absence of 200 nM P-stalk and 5 µM tRNA.

Figure S9. (A) eIF2α phosphorylated loop peptides. Mutated residues are indicated by darker font. **(B)** Coomassie stain, autoradiographs, and immunoblot with the indicated antibody of 30 min kinase assays with 20 nM GCN2, 200 nM P-stalk, and the indicated eIF2α peptides. **(C)** Coomassie stain, autoradiographs, and immunoblots with the indicated antibody of kinetic kinase assays with a titration of P-stalk as outlined in (Figure 6E). **(D)** Intensities of substrate autoradiographs

bioRxiv preprint doi: https://doi.org/10.64898/2026.09.09.750423; this version posted September 13, 2026. The copyright holder for this preprint (which was not certified by peer review) is the author/funder, who has granted bioRxiv a license to display the preprint in perpetuity. It is made available under a CC-BY 4.0 International license.

as in (B) plotted and fit with hypo-exponential functions. Error bars represent S.E.M. of 2 independent replicates. **(E)** V_0 in (D) as a function eIF2α peptide concentration fit with the Michaelis-Menten equation (see Methods). Error bars represent S.E.M. of 2 different replicates. Error on reported K_m is the standard error of the fit.

bioRxiv preprint doi: https://doi.org/10.64898/2026.09.09.750423; this version posted September 13, 2026. The copyright holder for this preprint (which was not certified by peer review) is the author/funder, who has granted bioRxiv a license to display the preprint in perpetuity. It is made available under a CC-BY 4.0 International license.

Supplementary Figures:

Figure S1

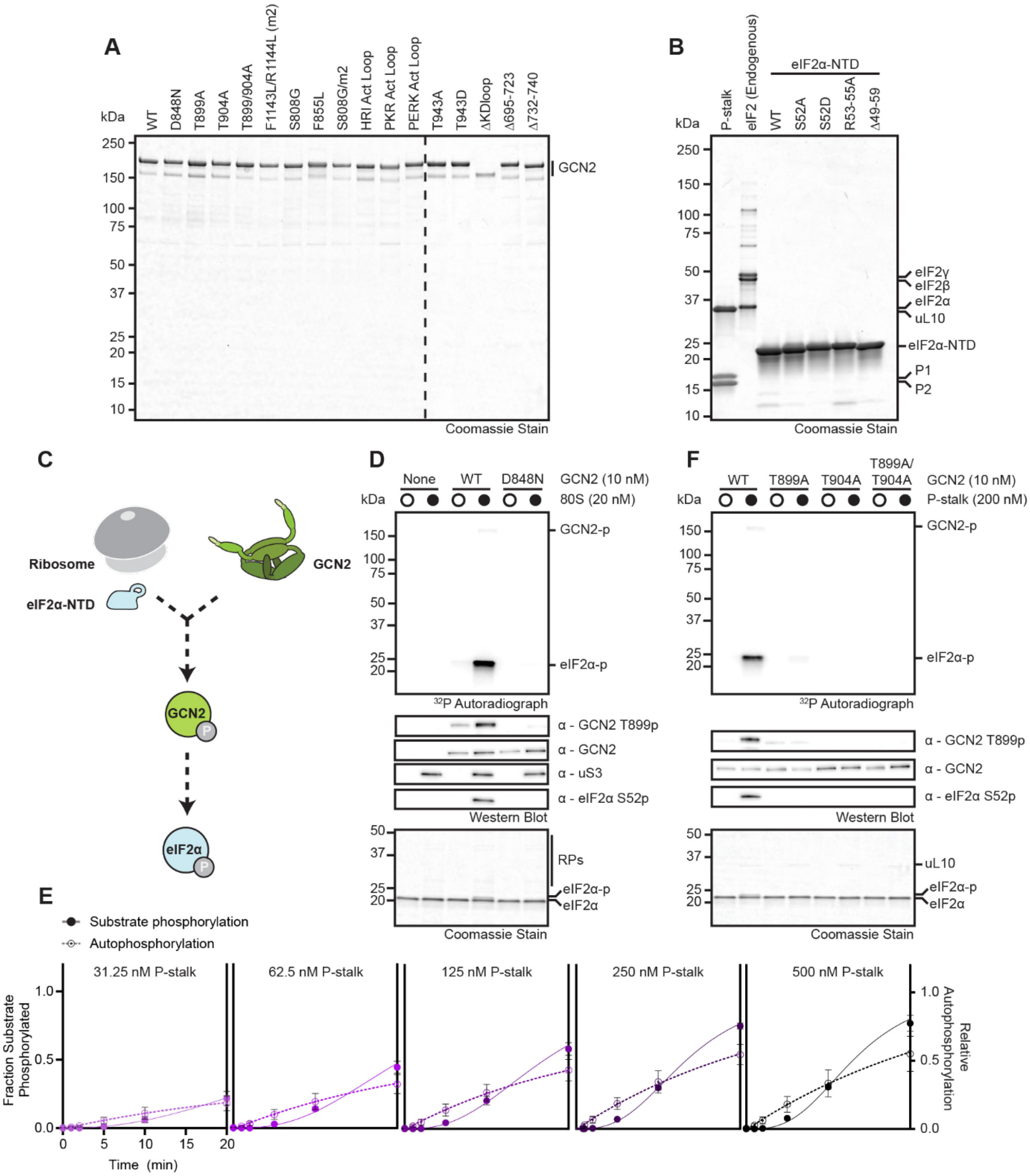

bioRxiv preprint doi: https://doi.org/10.64898/2026.09.09.750423; this version posted September 13, 2026. The copyright holder for this preprint (which was not certified by peer review) is the author/funder, who has granted bioRxiv a license to display the preprint in perpetuity. It is made available under a CC-BY 4.0 International license.

Figure S2

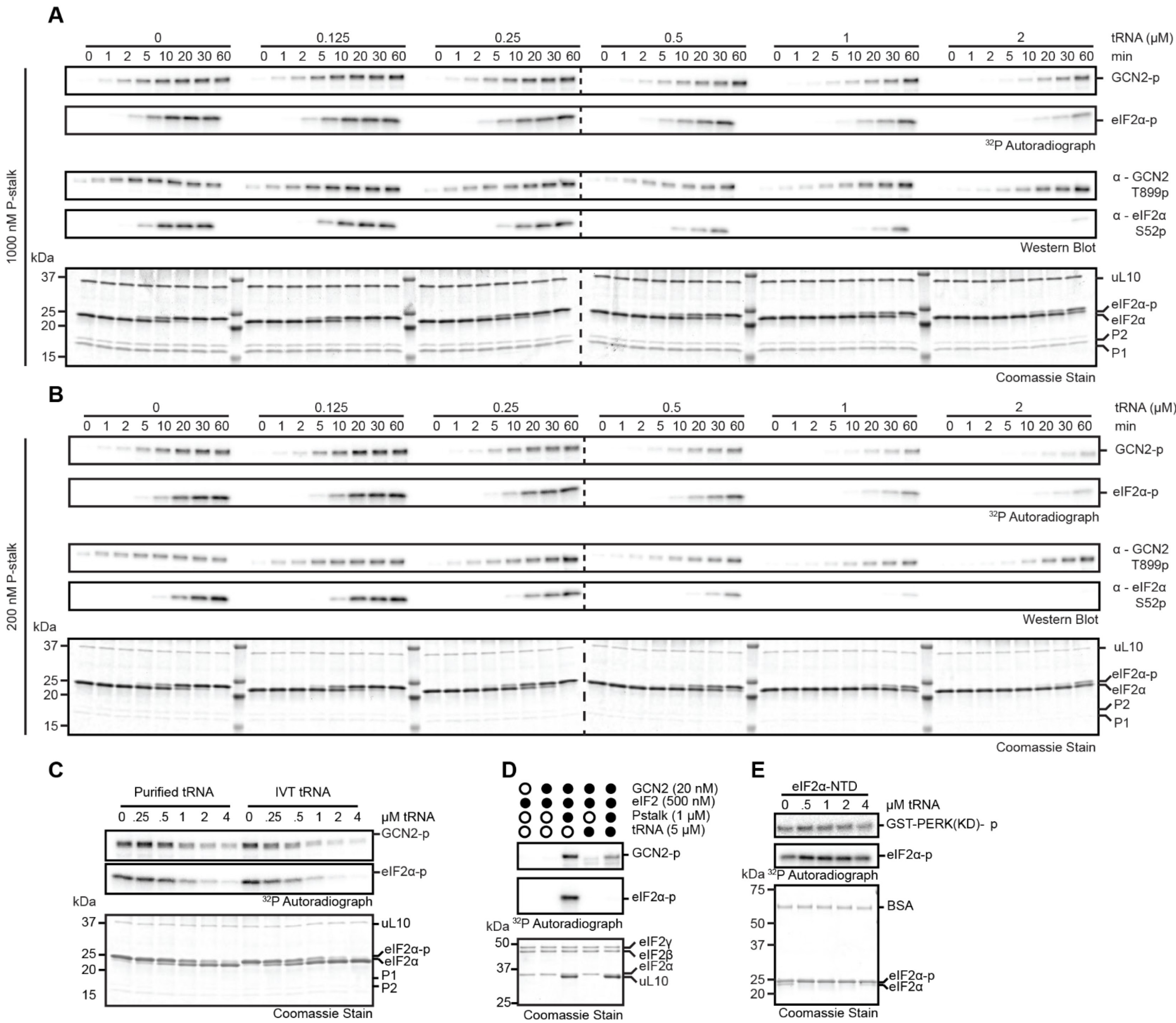

bioRxiv preprint doi: https://doi.org/10.64898/2026.09.09.750423; this version posted September 13, 2026. The copyright holder for this preprint (which was not certified by peer review) is the author/funder, who has granted bioRxiv a license to display the preprint in perpetuity. It is made available under a CC-BY 4.0 International license.

Figure S3

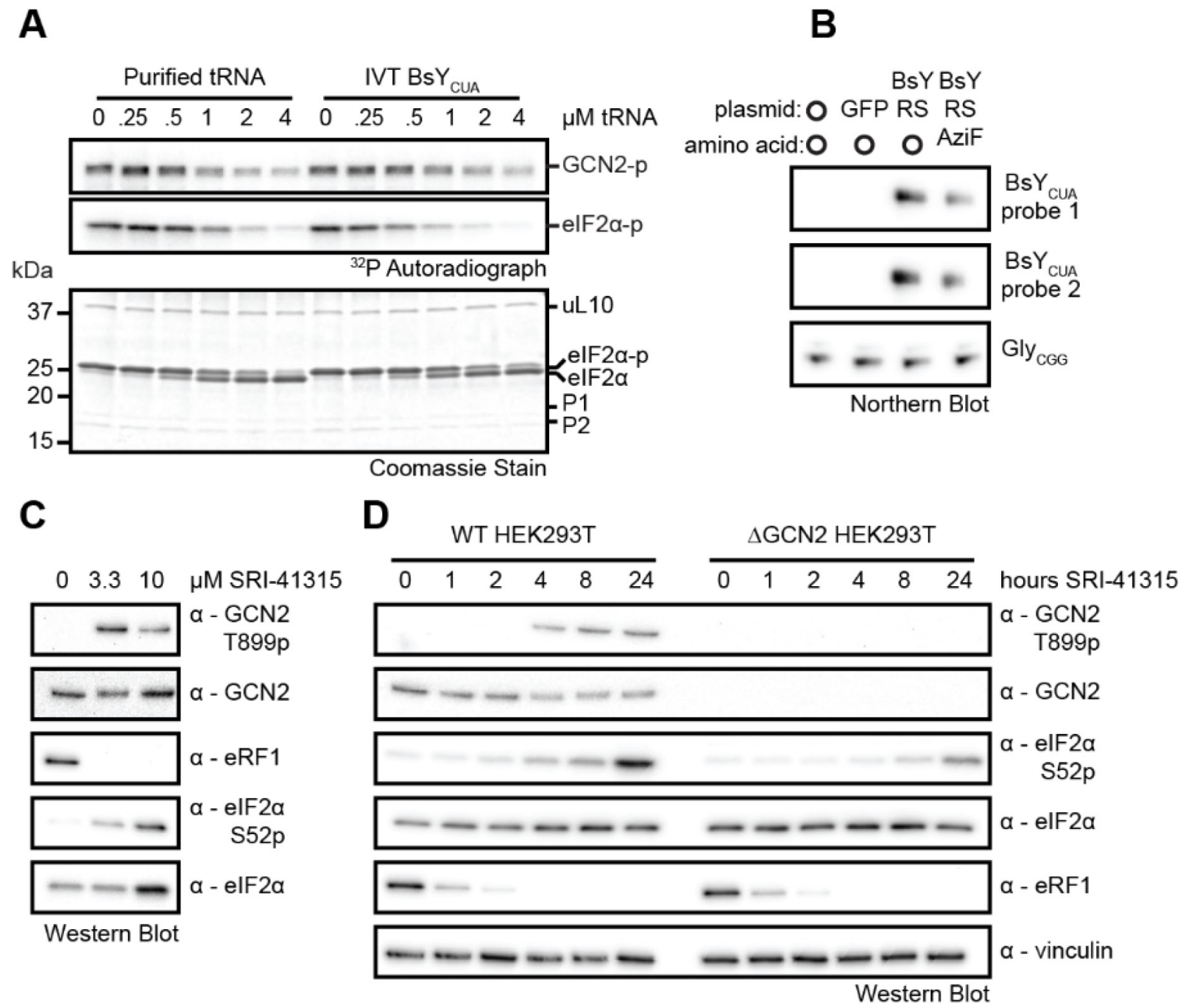

bioRxiv preprint doi: https://doi.org/10.64898/2026.09.09.750423; this version posted September 13, 2026. The copyright holder for this preprint (which was not certified by peer review) is the author/funder, who has granted bioRxiv a license to display the preprint in perpetuity. It is made available under a CC-BY 4.0 International license.

Figure S4

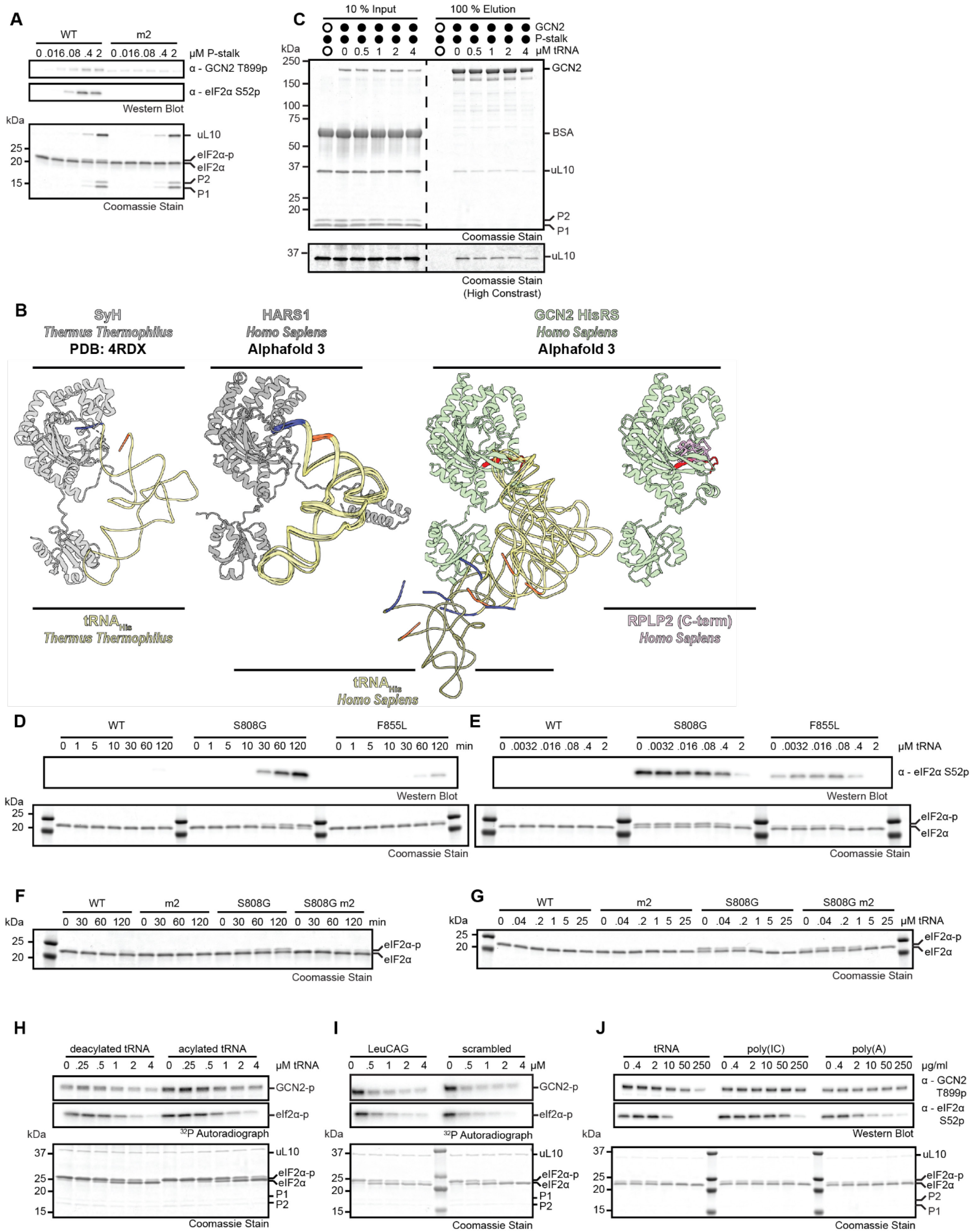

bioRxiv preprint doi: https://doi.org/10.64898/2026.09.09.750423; this version posted September 13, 2026. The copyright holder for this preprint (which was not certified by peer review) is the author/funder, who has granted bioRxiv a license to display the preprint in perpetuity. It is made available under a CC-BY 4.0 International license.

Figure S5

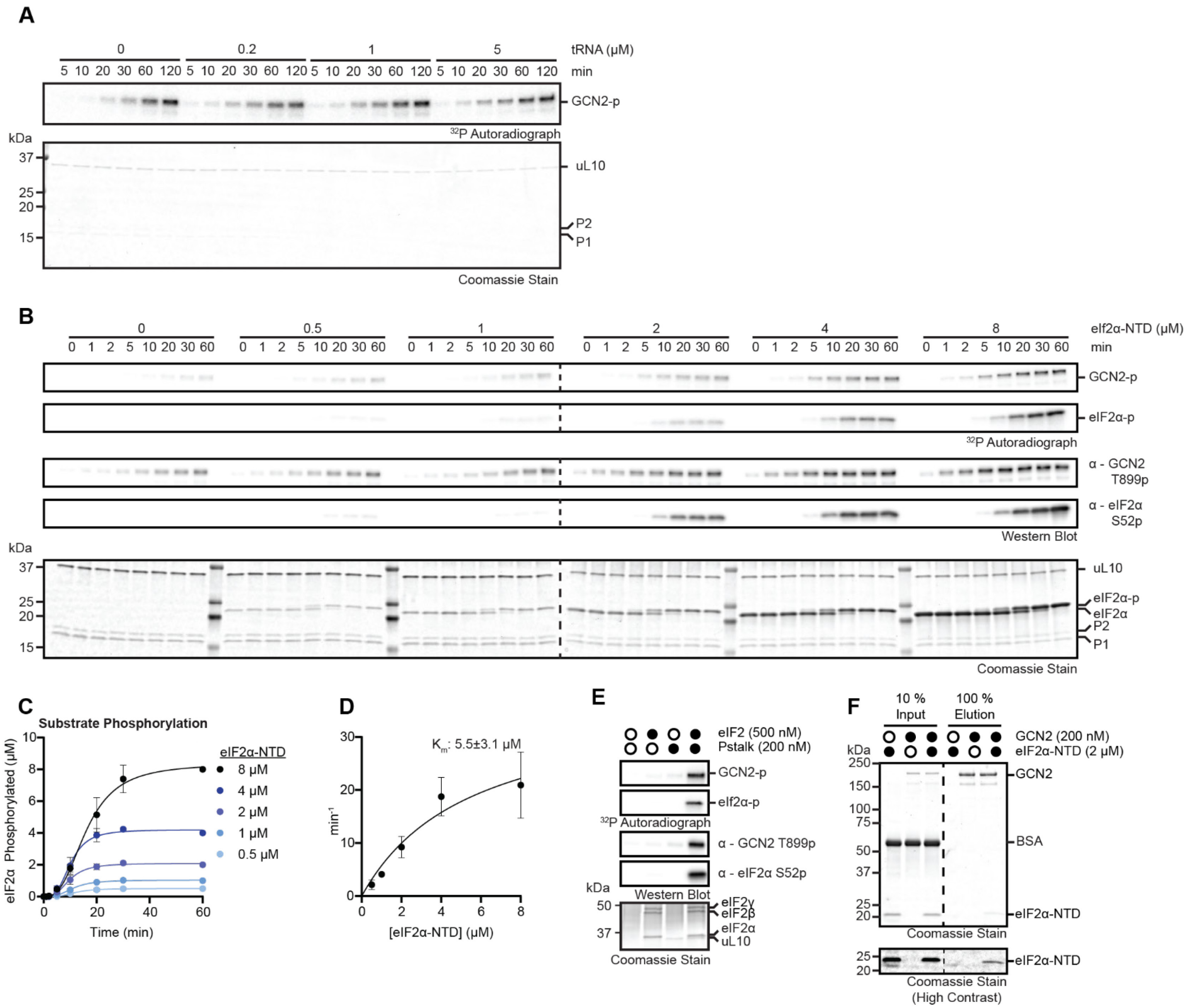

bioRxiv preprint doi: https://doi.org/10.64898/2026.09.09.750423; this version posted September 13, 2026. The copyright holder for this preprint (which was not certified by peer review) is the author/funder, who has granted bioRxiv a license to display the preprint in perpetuity. It is made available under a CC-BY 4.0 International license.

Figure S6

A

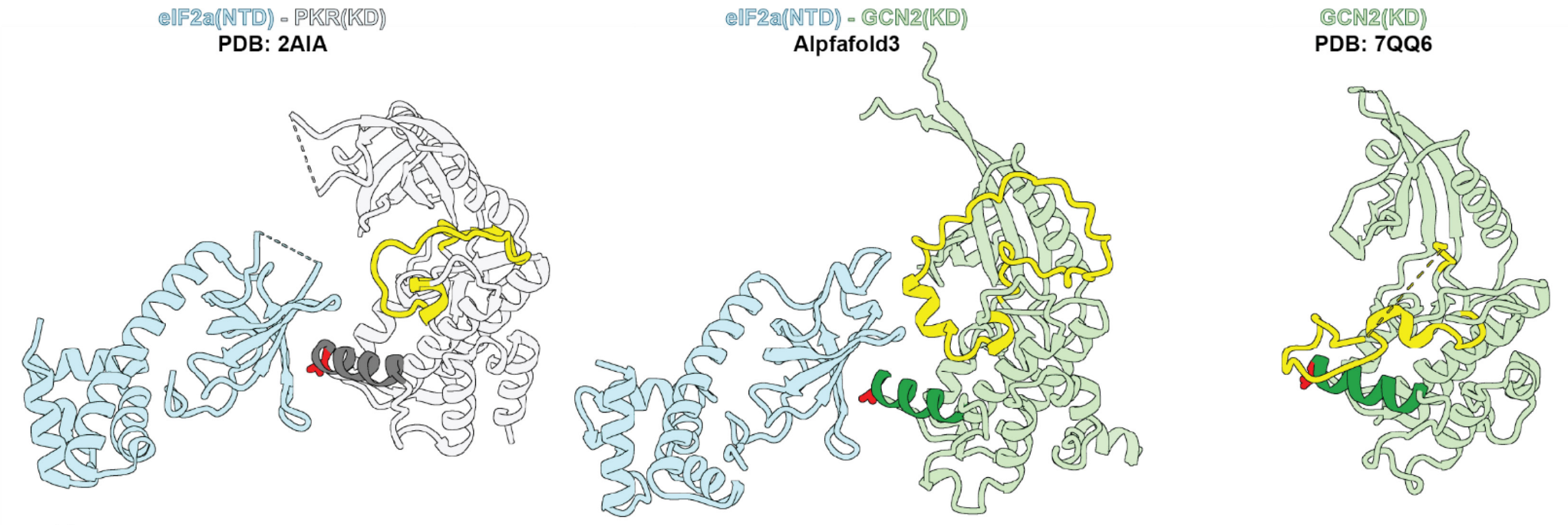

B

```
GCN2  866 DFGLATDHLAFSADSKQDDQTGDLIKSDPSGHLTGMVGTALYVSPEV 912
 HRI  461 DFGLACTDILQ------KNTDWTNRNGKRTPTHTSRVGTCLYASPEQ 501
 PKR  432 DFGLVTSLKND------GK-------------RTRSKGTLRYMSPEQ 459
PERK  955 DFGLVTAMDQD------EEEQTVLTPMPAYARHTGQVGTKLYMSPEQ 995
```

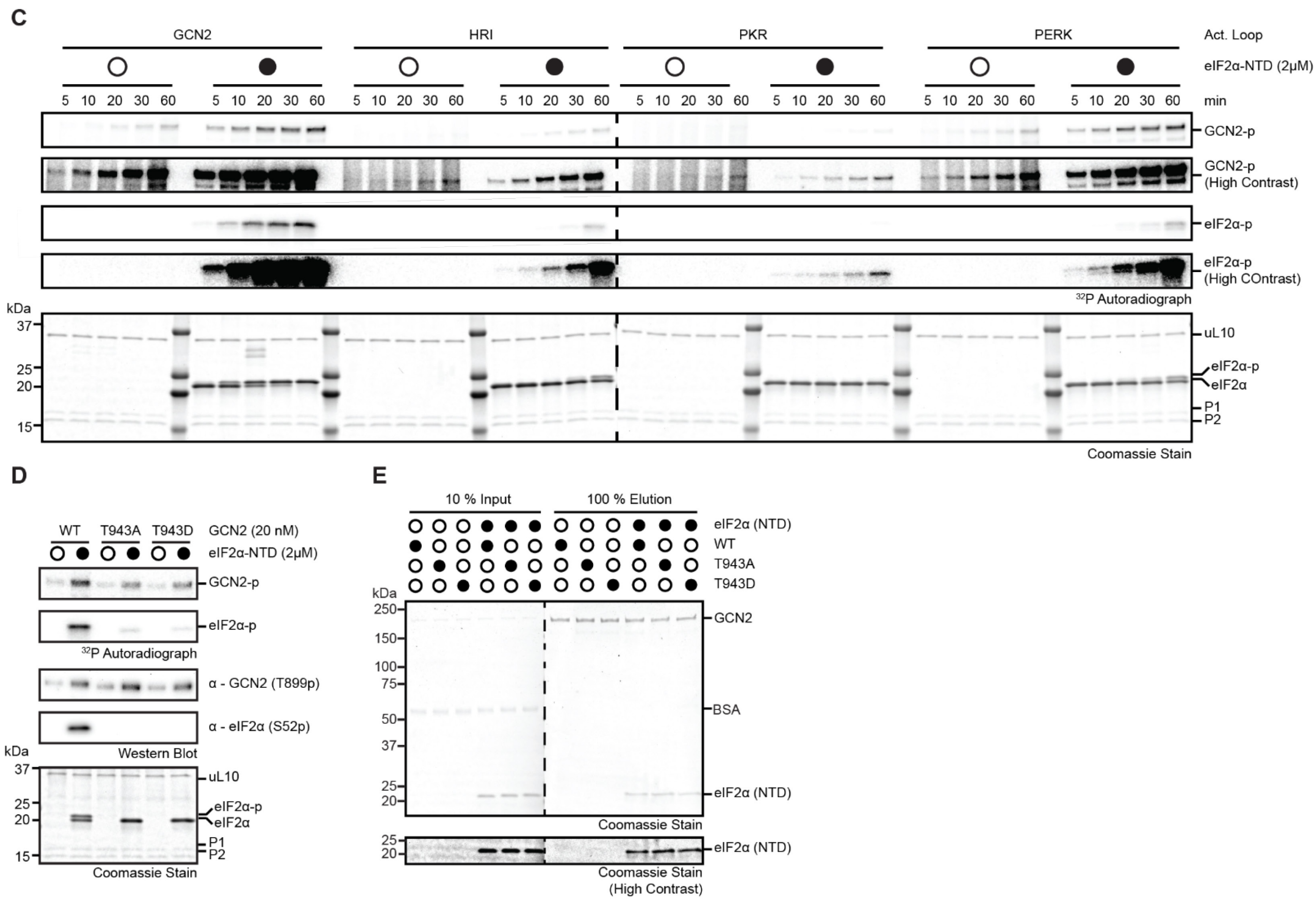

bioRxiv preprint doi: https://doi.org/10.64898/2026.09.09.750423; this version posted September 13, 2026. The copyright holder for this preprint (which was not certified by peer review) is the author/funder, who has granted bioRxiv a license to display the preprint in perpetuity. It is made available under a CC-BY 4.0 International license.

Figure S7

A

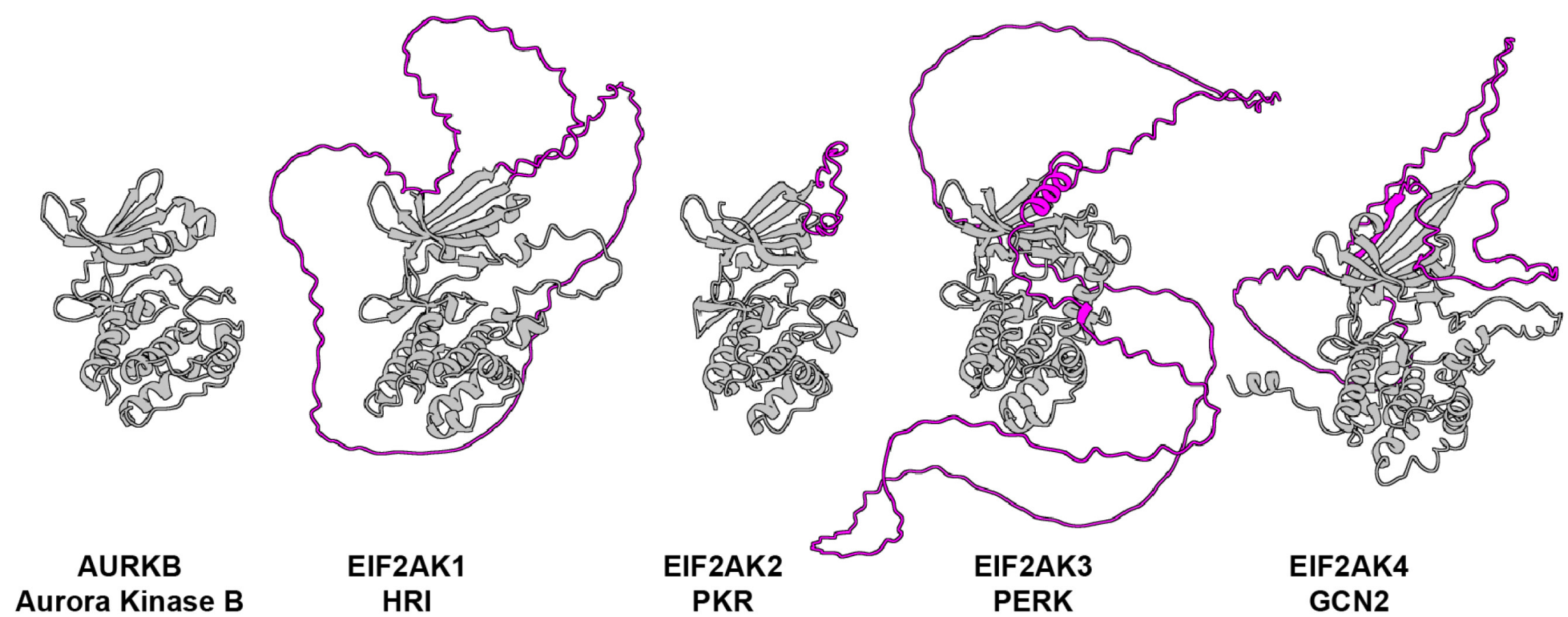

B

```
  AURKB/137-152 137 ILRLYNYFYD---------------------------------------------------- 146
EIF2AK1/226-382 226 IVGYHTAWIEHVHVIQ-PRADRAAI---------------ELPSLEVLSDQEEDREQCGVKN 271
EIF2AK2/320-364 320 IVHYNGCWDGFDYDPE---------------------------------------------T 336
EIF2AK3/651-886 651 IVRYFNAWLEAPPEKWQEKMDEIWLKDESTDWPLSSPSPMDAPSVKIR------RMDPF--A 704
EIF2AK4/648-800 648 IVRYYNAWIERHERPAGPGT----------------PPPDSGPLAKDD---RAARGQPA--S 688

  AURKB/137-152     --------------------------------------------------------------
EIF2AK1/226-382 272 DESSSSSIIFAEPTPEKEKRFG---------------------ESDTENQNNK-SVKYTT-- 309
EIF2AK2/320-364 337 S---DD-------------------------------------------------------- 339
EIF2AK3/651-886 705 T---KEHIEIIAPSPQRSRSFSVGISCDQTSSSESQFSPLEFSGMDHEDISESVDAAYNLQD 763
EIF2AK4/648-800 689 DTDGLDSVEAAAPPPILSSSVEWSTSGE--RSASARFPATGPGSSDDE-------------- 734

  AURKB/137-152     --------------------------------------------------------------
EIF2AK1/226-382 310 -----------------------------NLVIRESGELESTLELQENGLAGLSASSIVEQQ 342
EIF2AK2/320-364 340 -----------SL--------------------------------ES--------------- 343
EIF2AK3/651-886 764 SCLTDCDVEDGTMDGNDEGHSFELCPSEASPYVRSRERTSSSIVFEDSGCDNASSKEE-PKT 824
EIF2AK4/648-800 735 -----------DDDEDEHGGVFSQS------FLP-ASDSESDIIFDNEDENSKSQ------- 771

  AURKB/137-152 147 --------------------------------------------------------RRRIYL 152
EIF2AK1/226-382 343 LPLRRNSHLEE---SFTSTE--ESSEENV------------NF-----LGQTEAQYHLMLHI 382
EIF2AK2/320-364 344 -------------SDYDPENS----------------------------KNSSRSKTKCLFI 364
EIF2AK3/651-886 825 NRLHIGNHCANKLTAFKPTSSKSSSEATLSISPPRPTTLSLDLTKNTTEKLQPSSPKVYLYI 886
EIF2AK4/648-800 772 ---NQDEDCNEKNGC------------------------------HESEPSVTTEAVHYLYI 800
```

bioRxiv preprint doi: https://doi.org/10.64898/2026.09.09.750423; this version posted September 13, 2026. The copyright holder for this preprint (which was not certified by peer review) is the author/funder, who has granted bioRxiv a license to display the preprint in perpetuity. It is made available under a CC-BY 4.0 International license.

Figure S8

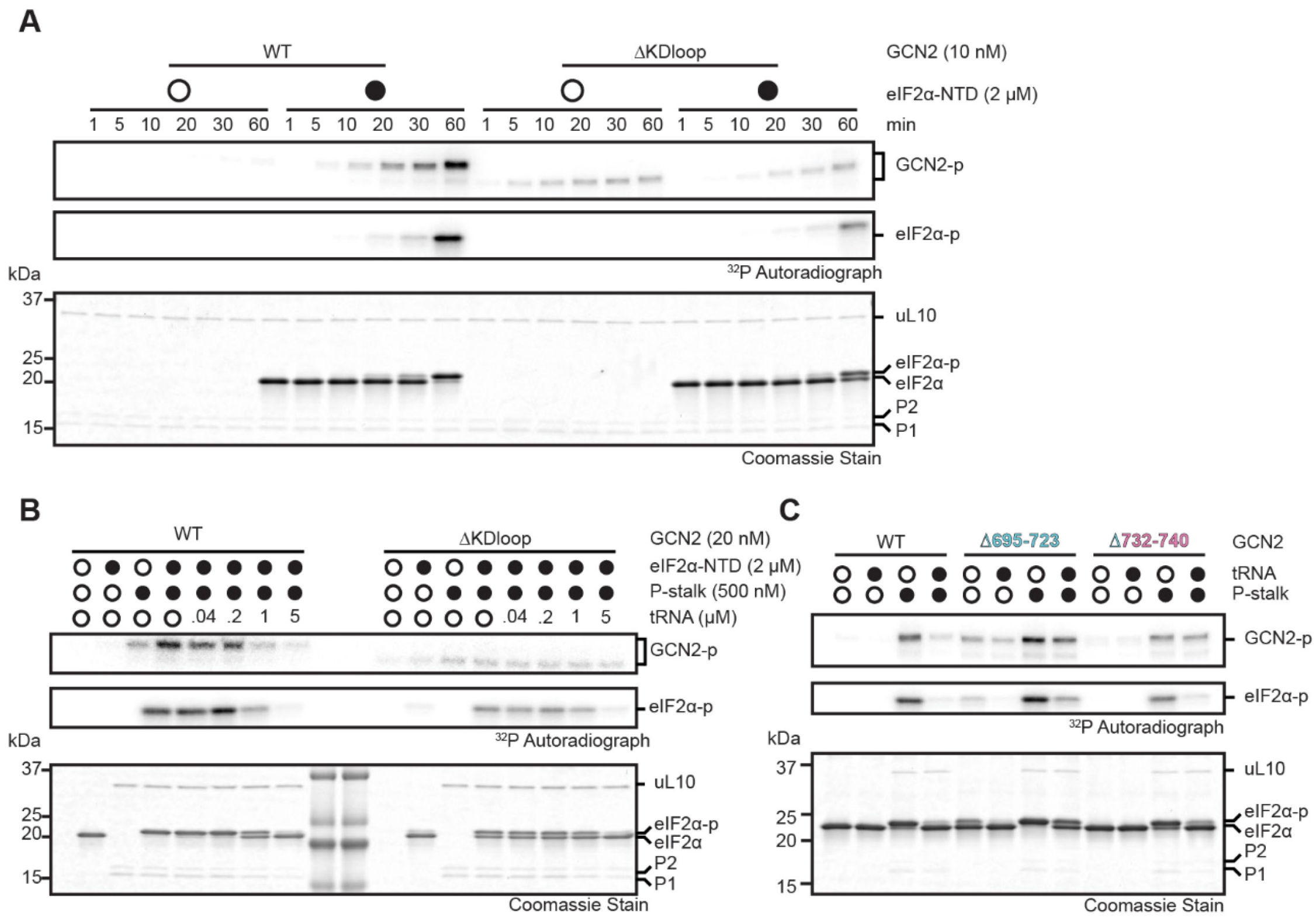

bioRxiv preprint doi: https://doi.org/10.64898/2026.09.09.750423; this version posted September 13, 2026. The copyright holder for this preprint (which was not certified by peer review) is the author/funder, who has granted bioRxiv a license to display the preprint in perpetuity. It is made available under a CC-BY 4.0 International license.

Figure S9

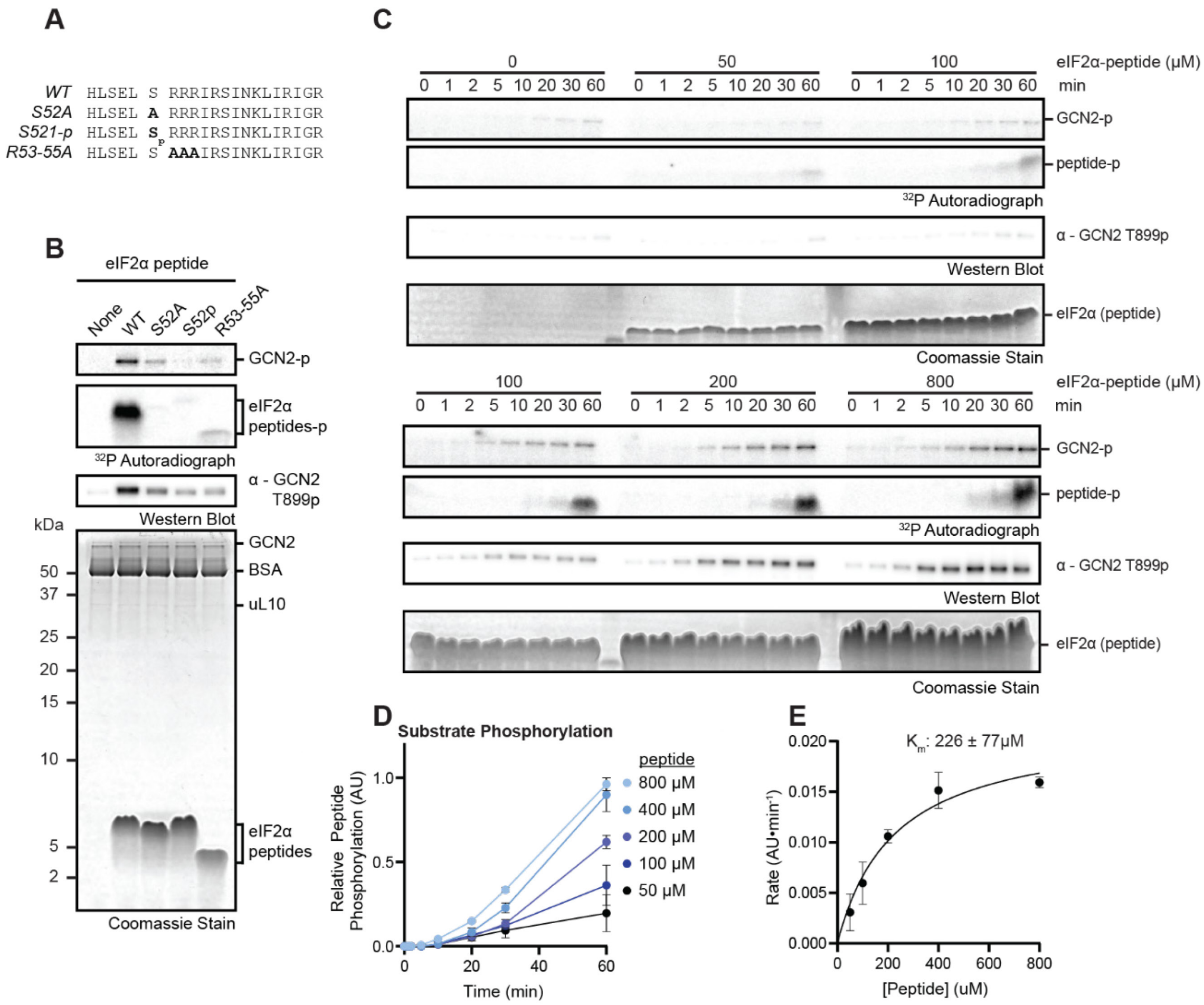

Supplement: Supplementary file 1 [file NIHPP2026.09.09.750423V1-supplement-1.pdf]
